# Fluoropyrimidine-associated gastrointestinal toxicity: pharmacovigilance reporting signals and *in vitro* evidence of epithelial injury, barrier impairment, and inflammatory responses

**DOI:** 10.3389/fimmu.2026.1934652

**Published:** 2026-09-29

**Authors:** Xinchi Luan, Xiaoran Li, Xiaoxuan Li, Xuezhe Wang, Xiaolei Zhang, Bangshen Zhao, Jia Xu, Zhanghao Yu, Guo Jing, Ning Liu

**Affiliations:** 1 Department of Oncology, The Affiliated Hospital of Qingdao University, Qingdao, Shandong, China; 2 Department of Cardiology, The Affiliated Hospital of Qingdao University, Qingdao, Shandong, China; 3 School of Basic Medicine, Qingdao University, Qingdao, Shandong, China

**Keywords:** epithelial injury, fluoropyrimidines, gastrointestinal adverse events, pharmacovigilance, reporting signals, time-to-onset

## Abstract

**Introduction:**

Fluoropyrimidines, including 5-fluorouracil (5-FU), capecitabine, tegafur, and trifluridine, are widely used anticancer agents, but their gastrointestinal toxicities remain clinically important. This study characterized fluoropyrimidine-associated adverse-event reporting signals across multiple pharmacovigilance databases and described epithelial-cell responses that may provide experimental context for gastrointestinal toxicity.

**Methods:**

Adverse-event reports from the US Food and Drug Administration Adverse Event Reporting System (FAERS), Japanese Adverse Drug Event Report database (JADER), and Canada Vigilance Adverse Reaction Database (CVARD) were analyzed using the reporting odds ratio (ROR), proportional reporting ratio (PRR), Bayesian confidence propagation neural network (BCPNN), and empirical Bayes geometric mean (EBGM) approaches. Time to onset (TTO) analysis was limited to FAERS because complete onset information was unavailable in JADER and CVARD. GES-1 gastric epithelial cells and NCM460 colonic epithelial cells were used for acute and 7-day repeated-exposure injury experiments. Additional representative-treatment experiments assessed fluorescein isothiocyanate (FITC)-dextran permeability and relative supernatant interleukin-6 (IL-6) and tumor necrosis factor alpha (TNF-α) levels.

**Results:**

Fluoropyrimidines showed gastrointestinal reporting signals with drug-specific reporting patterns. TTO analysis in FAERS showed an overall early-reporting pattern; the distribution of reports between days 181 and 360 was exploratory. *In vitro*, fluoropyrimidines reduced epithelial viability, increased lactate dehydrogenase (LDH) release and reactive oxygen species (ROS) accumulation and decreased the JC-1 red/green ratio. Epithelial injury was also observed under the 7-day repeated-exposure conditions. Quantitative real-time polymerase chain reaction (qPCR) showed lower *TJP1, OCLN*, and *CDH1* transcript abundance and increased expression of selected epithelial inflammatory-response and extracellular-matrix-remodeling genes. In the functional assays, FITC-dextran permeability and relative supernatant IL-6 and TNF-α levels increased after 5-FU, capecitabine, and trifluridine exposure.

**Conclusion:**

This study identifies pharmacovigilance reporting signals and provides experimental evidence that fluoropyrimidine exposure is associated with epithelial injury, impaired barrier function, and epithelial inflammatory responses. The reporting signals do not estimate event frequency in treated populations or establish causality and require confirmation in exposure-adjusted studies.

## 1 Introduction

Fluoropyrimidines, including 5-fluorouracil (5-FU), capecitabine, tegafur, and trifluridine (clinically administered with tipiracil), are cornerstone therapies for multiple solid tumors (1–3). Their antimetabolite effects also contribute to clinically important toxicity. Registration trials may incompletely characterize rare events, heterogeneous populations, and events reported outside protocol-defined observation windows (4–6). Post-marketing analyses can therefore identify reporting signals that merit further study, but proportions of fatal outcomes among submitted reports do not estimate fatality among treated patients (7, 8).

Spontaneous reporting systems (SRS) complement trial data through broad post-marketing coverage, but they lack reliable exposure denominators and are affected by underreporting, stimulated reporting, duplicate or follow-up reports, indication and notoriety biases, heterogeneous market use, and missing clinical information (9, 10). Cross-database differences in report counts or demographic proportions therefore describe submitted reports and cannot establish geographic, biological, sex-specific, or body-weight-related susceptibility.

We analyzed the US Food and Drug Administration Adverse Event Reporting System (FAERS), Japanese Adverse Drug Event Report database (JADER), and Canada Vigilance Adverse Reaction Database (CVARD) spanning 1990-2024 (11–14). Four correlated disproportionality methods—the reporting odds ratio (ROR), proportional reporting ratio (PRR), Bayesian confidence propagation neural network (BCPNN), and empirical Bayes geometric mean (EBGM)—plus FAERS time-to-onset analysis, and clinical-priority scoring were used to characterize reporting patterns (15, 16). The objectives were to identify reporting signals, describe their consistency across heterogeneous reporting systems, and provide epithelial-cell context; the study was not designed to estimate event frequency in treated populations, prove causality, or support regulatory action.

## 2 Materials and methods

### 2.1 Data sources and processing

We performed a retrospective analysis of pharmacovigilance utilizing the FAERS database (17), JADER database and CVARD database. The US Food and Drug Administration (FDA) maintains the Adverse Event Reporting System (FAERS), a database that stores data regarding adverse events and medication errors reported to the agency. The dataset encompasses seven distinct data tables, patient demographic and administrative details (DEMO), information on drugs and biologics (DRUG), adverse events (REAC), patient outcomes (OUTC), sources of reports (RPSR), drug therapy start and end dates (THER), and indications for use or diagnoses (INDI). These tables collectively provide comprehensive information for analysis. This system is specifically designed to assist the FDA in conducting post-marketing safety surveillance for drugs and therapeutic biologics. With over 4 million voluntarily submitted reports, FAERS is recognized as the world’s largest collection of spontaneously reported adverse event data.

The Japanese Adverse Drug Event Report (JADER), established in 2004, is composed of four main files: DEMO, DRUG, REAC, and medical history (HIST). These files collectively store detailed information on cases reported by pharmaceutical companies and medical institutions. The DEMO file features basic patient information, including sex, age, and weight. The DRUG file details the generic name of the medication, the route of administration, and the dates when the drug was administered. The REAC file documents the name of the adverse event, its outcome, and the date it occurred. Meanwhile, the HIST file provides information about the patient’s underlying medical conditions. Together, these files enable comprehensive monitoring and analysis of adverse events associated with medications.

The Canadian Vigilance Adverse Reaction Database (CVARD), established in 1965, comprises reports submitted by healthcare professionals, consumers, manufacturers, and distributors. The database is managed by seven regional offices under the Canadian Vigilance Program, which oversees its operation. This program employs the database to conduct post-market safety monitoring of medications, ensuring their safety following market release.

For each database, analysis-specific denominators were derived from raw downloaded records, duplicate removal, unique case consolidation, target-drug identification, primary-suspect restriction, drug-event pair construction, and Medical Dictionary for Regulatory Activities (MedDRA) preferred-term mapping (**Figures 1A–C**).

**Figure 1:**
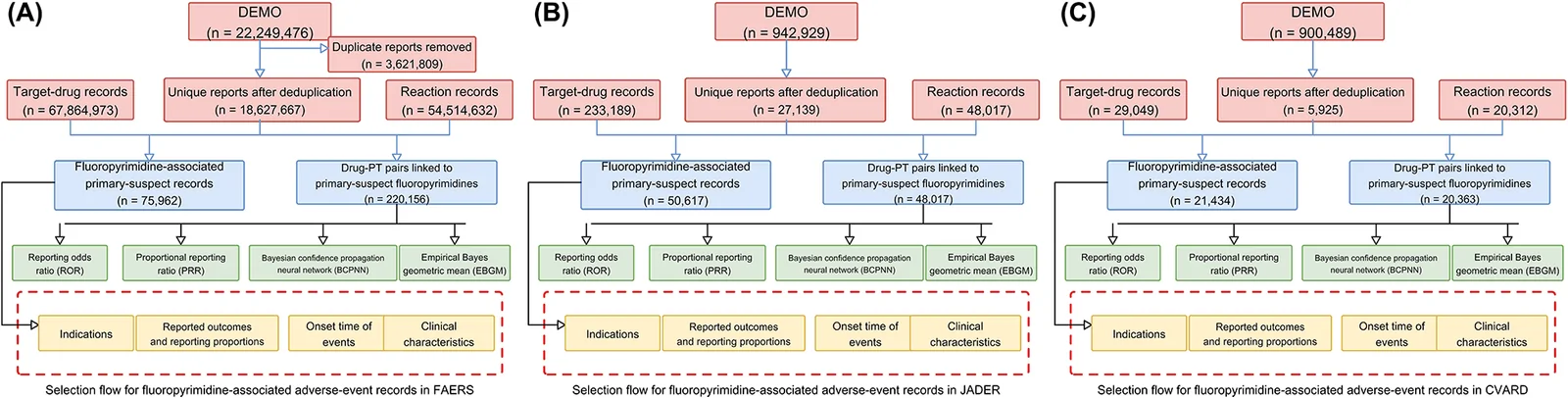
Selection of fluoropyrimidine-associated adverse-event records across the three pharmacovigilance databases. **(A)** FAERS selection flow. **(B)** JADER selection flow. **(C)** CVARD selection flow. The boxes show database records retained at successive stages of duplicate removal, target-drug identification, primary-suspect restriction, reaction linkage, and preferred-term mapping. The unique-report denominators used for the demographic analyses were 75,962 for FAERS, 27,139 for JADER, and 5,925 for CVARD. Counts of linked drug records, reaction records, and drug–PT pairs may exceed the corresponding unique-report denominators because an individual report may contain multiple drugs or adverse events. AE, adverse event; PT, preferred term; PS, primary suspect; FAERS, US food and drug administration event reporting system; JADER, Japanese adverse drug event report database; CVARD, Canada vigilance adverse reaction database.

### 2.2 Drug standardization and adverse event classification

Drug names were standardized using MedEx Unstructured Information Management Architecture (UIMA) 1.3.8, and adverse events were mapped using MedDRA version 26.1. A report denotes one submitted safety-report record after the verified database-specific follow-up rule; a unique case denotes the consolidated patient report; a preferred term (PT) denotes a MedDRA-coded event; and a drug-event pair denotes one eligible target-drug/PT combination. Target-drug analyses were restricted to records coded as primary suspect.

### 2.3 Signal detection

Disproportionality was assessed using ROR, PRR, BCPNN, and EBGM as correlated signal-detection approaches rather than independent validations. For each database, reports involving a target fluoropyrimidine were compared with reports involving all other drugs in that database using the 2 × 2 framework defined in **Supplementary Table S1**. **Supplementary Table S2** provides the formulas, confidence intervals, and prespecified signal criteria. No multivariable adjustment was performed; analyses by age, sex, weight, and report source were therefore treated as exploratory stratified analyses.

### 2.4 Clinical significance analysis

Adverse events (AEs) with significant adverse reactions were prioritized based on clinical significance as follows:

First, important medical events (IMEs) and designated medical events (DMEs) were identified using the public list developed and updated by the European Medicines Agency. IMEs are adverse events with features of seriousness, whereas DMEs are rare, severe events that may be plausibly drug related and warrant focused safety review.

Second, IMEs and DMEs were prioritized using a semi-quantitative score comprising reporting proportion, consistency across the four disproportionality methods, reported case-fatality proportion, and serious-event classification (**Supplementary Table S3**). Scores of 0–2, 3–5, or 6–8 were categorized as weak, moderate, or strong reporting-signal priority, respectively. This prioritization supports signal review and does not establish a clinical-practice recommendation.

### 2.5 Time to onset analysis

Time to onset (TTO) was calculated as the interval between treatment initiation (START_DT) and event onset (EVENT_DT). Only FAERS records containing complete calendar dates for both variables were included. Records with missing or incomplete dates, same-day intervals (TTO = 0), or event dates preceding treatment initiation were excluded, and missing dates were not imputed. Because an individual FAERS report could contain more than one qualifying drug-start record, the numbers of unique evaluable reports and evaluable TTO records were documented separately. Drug-specific median TTO values and interquartile ranges (IQRs) were calculated. Weibull distributions were fitted by maximum likelihood estimation, and the scale parameter (α), shape parameter (β), and corresponding 95% confidence intervals (CIs) were estimated. A β value below 1 was interpreted as an early-reporting pattern, β equal to 1 as a constant reporting pattern, and β above 1 as an increasing reporting pattern. A sensitivity analysis excluded five extreme TTO records exceeding 10,000 days that were considered likely date-entry anomalies. TTO analysis was limited to FAERS because complete onset-date information was unavailable in JADER and CVARD.

### 2.6 Subgroup analyses

Exploratory sex-stratified analyses were performed at the system organ class (SOC) and PT levels. Because sex-specific data were unavailable in CVARD, no such analysis was performed for that database. These comparisons describe submitted-report patterns and do not estimate sex-specific susceptibility.

### 2.7 *In vitro* epithelial-cell experiments

Human GES-1 gastric epithelial cells were purchased from IMMOCELL (Xiamen, Fujian, China), and human NCM460 colonic epithelial cells were purchased from IMMOCELL (Xiamen, Fujian, China). GES-1 cells were maintained in Dulbecco’s Modified Eagle Medium (DMEM, High glucose, PM150210; Wuhan Procell Biotechnology Co., Ltd.) supplemented with 10% fetal bovine serum (FBS; XRFBS10-500; XR Biotechnolgies Co., Ltd., Hangzhou, China) and 1% penicillin–streptomycin solution (BC-CE-007; Senbeijia Biological Technology Co., Ltd.), whereas NCM460 cells were maintained in RPMI-1640 medium(AC01L064; Shanghai Life-iLab Biotech Co., Ltd). Cells were maintained at 37°C in a humidified incubator containing 5% CO2.For subculture, the culture medium was removed, and the cell monolayer was washed once with phosphate‑buffered saline (PBS). Then, 2 mL of 0.25% trypsin–EDTA solution (SB-CR013N; Share-bio, Shang Hai,China) was added to each 10‑cm culture dish (H802002; BaiDi Biotechnology Co., Ltd. (BDBIO)), and the cells were incubated at 37 °C for 2–3 min until they detached. Trypsinization was neutralized by adding an equal volume of complete culture medium containing 10% FBS. The cell suspension was transferred to a 15‑mL centrifuge tube and centrifuged at 1,000 rpm for 5 min at room temperature. The supernatant was discarded, and the cell pellet was resuspended in fresh complete medium for further passage or in a serum‑free cell cryopreservation medium (AC05L033; Shanghai Life-iLab Biotech Co., Ltd) for long‑term storage. For cryopreservation, the cell density was adjusted to approximately 1–2 × 10^6^ cells/mL in the cryopreservation medium, and 1‑mL aliquots were dispensed into sterile cryogenic vials (AC05-20, BIOLAND). The vials were placed in a controlled‑rate freezing container and stored at –80 °C overnight, then transferred to liquid nitrogen for long‑term maintenance.

5-Fluorouracil (5-FU,TargetMol, USA, T0984), capecitabine (TargetMol, USA,T1408), and trifluridine (T70086,Psaitong Biotechnology, Beijing,China.) were dissolved in dimethyl sulfoxide (DMSO) to prepare stock solutions. The final solvent concentration was identical across treatment groups and did not exceed 0.1% (v/v). For acute-exposure experiments, cells were treated with 50 μM 5-FU, 100 μM capecitabine, or 50 μM trifluridine for 24 h. For repeated-exposure experiments, cells were exposed to 10 μM 5-FU, 20 μM capecitabine, or 10 μM trifluridine for 7 days, with fresh medium and drug replacement on days 3 and 5. Vehicle-control cultures received the corresponding concentration of DMSO. The repeated-exposure model was used to evaluate epithelial responses to sustained drug exposure and was not intended to reproduce clinical events reported 181–360 days after treatment initiation (**Figure 2A**).

**Figure 2:**
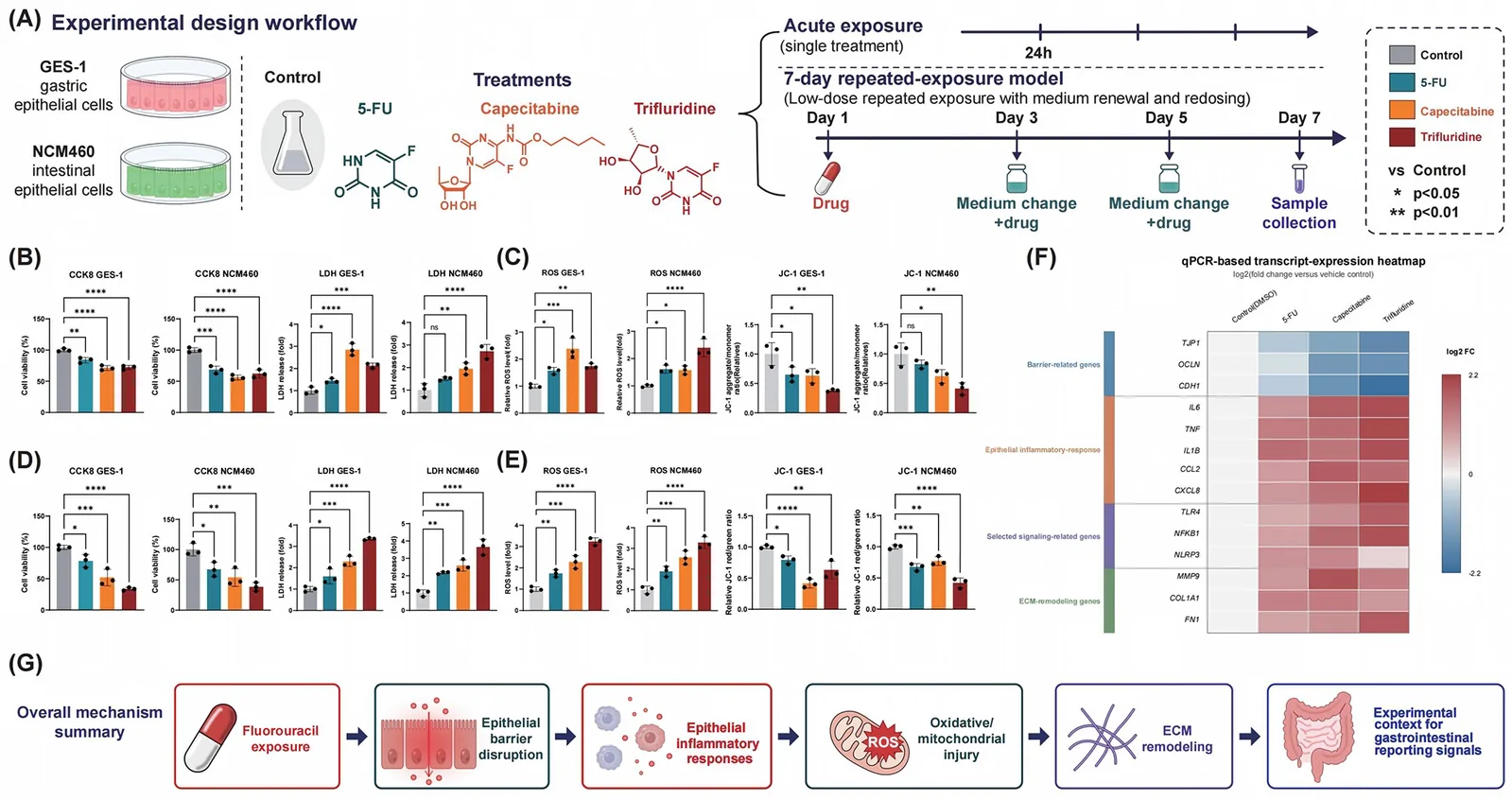
*In vitro* epithelial responses to fluoropyrimidine exposure under acute and repeated-exposure conditions. **(A)** Experimental workflow for acute exposure and the 7-day repeated-exposure model in GES-1 and NCM460 cells. **(B)** Cell viability and lactate dehydrogenase release after acute exposure. **(C)** Reactive oxygen species levels and JC-1 red/green ratios after acute exposure. **(D, E)** Cell viability, lactate dehydrogenase release, reactive oxygen species levels, and JC-1 red/green ratios after repeated exposure. **(F)** qPCR-based heatmap showing relative transcript abundance of barrier-related genes, epithelial inflammatory-response genes, selected signaling-related genes, and extracellular-matrix-remodeling genes. Gene symbols represent mRNA targets and do not indicate protein abundance. **(G)** Schematic summary of the observed epithelial responses. Data are presented as mean ± SD from three independent experiments. Groups were compared using ordinary one-way ANOVA followed by Dunnett’s multiple-comparison test versus vehicle control.

Cell viability was measured using a Cell Counting Kit-8 assay (CCK-8; CA1212; Solarbio Science & Technology Co., Ltd., Beijing, China) according to the manufacturer’s instructions. Lactate dehydrogenase release was measured in culture supernatants using an LDH cytotoxicity assay kit (G0804F,Suzhou Grace Biotechnology Co.,Ltd). Intracellular reactive oxygen species were assessed using a DCFDA/2′,7′-dichlorodihydrofluorescein diacetate (H_2_DCFDA) - Cellular ROS Assay Kit (Abcam, Cambridge, UK, catalog no. ab113851), with fluorescence measured at excitation/emission wavelengths of 485 nm/535 nm. Mitochondrial membrane potential was evaluated using a JC-1 Mitochondrial Membrane Potential Assay Kit (MedChemExpress, catalog no. HY-K0601). Red and green fluorescence signals were measured at excitation/emission wavelengths of 510/527 nm (green, JC-1 monomers) and 585/590 nm (red, JC-1 aggregates), and the red-to-green fluorescence ratio was calculated. A lower ratio was interpreted as mitochondrial depolarization (**Figures 2B–E**).

Total RNA was extracted using TRIzol Reagent (CW0580, CWBIO,China). RNA concentration and purity were determined using a NanoDrop 2000 spectrophotometer (Thermo Fisher Scientific).

Complementary DNA was synthesized using the Hifair^®^ III 1st Strand cDNA Synthesis Kit (11141ES60; Yeasen Biotechnology Co., Ltd., Shanghai, China). Quantitative real-time PCR was then performed using UltraStart SYBR Green qPCR Master Mix (A403; EXONGEN (Chengdu, China)) on a QuantStudio™ 5 Real-Time PCR System (Thermo Fisher Scientific).

Quantitative real-time polymerase chain reaction (qPCR) was used to quantify transcripts associated with epithelial barrier integrity (*TJP1, OCLN, and CDH1*), epithelial inflammatory responses (*IL6, TNF, IL1B, CXCL8, and CCL2*), inflammation-related signaling (*TLR4, NFKB1, and NLRP3*), and extracellular matrix remodeling (*MMP9, COL1A1, and FN1*) (**Figure 2F**). Because qPCR measures transcript abundance, these findings were interpreted as epithelial transcriptional responses rather than direct evidence of altered junctional protein abundance or immune-cell activation. Functional barrier integrity was evaluated separately using the FITC-dextran permeability assay described below.

### 2.8 FITC-dextran permeability assay

Functional epithelial permeability was evaluated in confluent NCM460 and GES-1 monolayers cultured on Transwell inserts (NEST Biotechnology, Wuxi, China; catalogue number 725121; pore size, 0.4 μm; membrane area, 0.33 cm^2^). Cells were seeded at 1 × 10^5^ cells per insert and cultured until a confluent monolayer was obtained. Monolayers were then exposed to vehicle, 10 μM 5-FU, 20 μM capecitabine, or 10 μM trifluridine for 7 days.

After treatment, the apical compartment was incubated with 40-kDa fluorescein isothiocyanate-dextran (FITC-dextran; FD40, Sigma-Aldrich, St. Louis, MO, USA) at a final concentration of 1 mg/mL. After 60 min incubation at 37 °C, samples were collected from the basolateral compartment, and fluorescence was measured at excitation and emission wavelengths of 485 and 530 nm, respectively, using a microplate reader (Tecan Infinite M200 PRO, Tecan Group Ltd., Männedorf, Switzerland). Permeability was expressed relative to the vehicle-control group. Three independent experiments were performed for each treatment group (**Figure 3A**).

**Figure 3:**
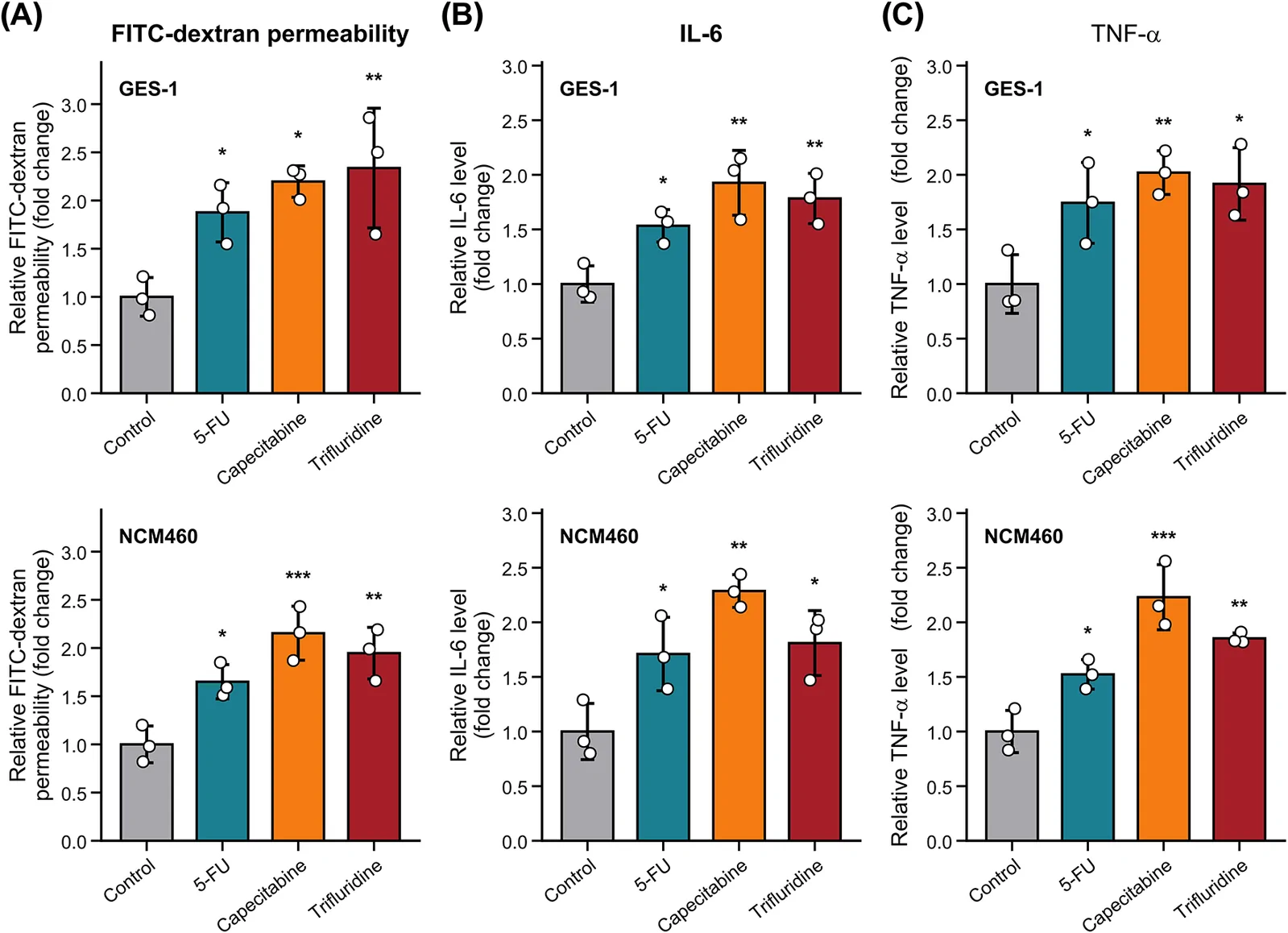
Fluoropyrimidine-associated increases in epithelial permeability and cytokine release. **(A)** Relative FITC-dextran permeability in GES-1 and NCM460 epithelial monolayers. **(B)** Relative IL-6 levels in culture supernatants. **(C)** Relative TNF-α levels in culture supernatants. Data are presented as mean ± SD from three independent experiments; points represent independent experiments. Comparisons were performed using ordinary one-way ANOVA followed by Dunnett’s multiple-comparison test versus vehicle control. *P < 0.05, **P < 0.01, ***P < 0.001.

### 2.9 Enzyme-linked immunosorbent assay

Culture supernatants were collected from NCM460 and GES-1 cells after exposure to vehicle, 10 μM 5-FU, 20 μM capecitabine, or 10 μM trifluridine for 7 days. Supernatants were centrifuged at 300 × g for 10 min at 4 °C to remove cellular debris and were analyzed immediately or stored at −80 °C until analysis.

Interleukin-6 and tumor necrosis factor alpha concentrations were measured using human IL-6 (NME3186HN,orminkoda Biotechnology Co., Ltd. (Wuhan, China)) and human TNF-α (ORSE3738H,AOPEISAI Co., Ltd. (Shanghai, China)) enzyme-linked immunosorbent assay kits according to the manufacturers’ instructions. Absorbance was measured at 450 nm using a microplate reader (Tecan Infinite M200 PRO, Tecan Group Ltd., Männedorf, Switzerland). Cytokine concentrations were calculated from kit-specific standard curves and expressed relative to the vehicle-control group. Three independent experiments were performed for each treatment group (**Figures 3B, C**).

### 2.10 Statistical analysis

Pharmacovigilance analyses were performed using the database-specific methods described above. *In vitro* data are presented as mean ± standard deviation (SD) from three independent experiments. Multiple-group comparisons were performed using ordinary one-way analysis of variance (ANOVA) followed by Dunnett’s multiple-comparison test, with each fluoropyrimidine group compared with the vehicle control. Brown-Forsythe testing did not indicate unequal variances for the FITC-dextran, IL-6, or TNF-α datasets. Two-sided adjusted P < 0.05 was considered statistically significant. Data management used Microsoft Excel 2019, and statistical analysis and visualization used R version 4.4.1 and GraphPad Prism 10.1.2.

## 3 Results

### 3.1 Descriptive characteristics

The analysis-specific selection pathways for FAERS, JADER, and CVARD are summarized in **Figures 1A–C**. After duplicate removal and case consolidation, the demographic analyses included 75,962 unique FAERS reports, 27,139 unique JADER reports, and 5,925 unique CVARD reports (**Table 1**). The larger downstream counts shown in some **Figure 1** boxes represent linked primary-suspect drug records, reaction records, or drug–PT pairs rather than additional unique reports. The observation periods were 2004–2024 for FAERS and JADER and 1990–2023 for CVARD.

In FAERS, documented-sex counts were internally consistent (female, 39,190; male, 30,097; unknown, 6,675). Adults aged 18–64 years formed the largest age group. Weight was recorded in 20,714 reports (27.3%), including 1,804 reports for patients weighing <50 kg, 17,130 for those weighing 50–100 kg, and 1,780 for those weighing ≥100 kg (**Table 1**).

**Table 1:** Demographic characteristics of fluoropyrimidine-associated reports in the FAERS, JADER, and CVARD databases.

Database	FAERS	JADER	CVARD
Report count	75962	27139	5925
Sex			
Female	39190 (51.6%)	9754 (35.9%)	2454 (41.4%)
Male	30097 (39.6%)	15583 (57.4%)	1566 (26.4%)
Unknown	6675 (8.8%)	1802 (6.6%)	1905 (32.2%)
Age			
<18	288 (0.4%)	31 (0.11%)	14 (0.2%)
>85	1172 (1.5%)	265 (0.98%)	46 (0.8%)
18–64	27067 (35.6%)	15332 (56.49%)	2787 (47.0%)
65–85	23333 (30.7%)	10038 (36.99%)	2211 (37.3%)
Unknown	24102 (31.7%)	1473 (5.43%)	867 (14.6%)
Weight			
<50 kg	1804 (2.4%)	10426 (38.42%)	85 (1.4%)
≥100 kg	1780 (2.3%)	16 (0.06%)	88 (1.5%)
50–100 kg	17130 (22.6%)	4522 (16.66%)	1205 (20.4%)
Unknown	55248 (72.7%)	12175 (44.86%)	4547 (76.7%)

In JADER, documented-sex and age counts were internally consistent, with adults aged 18–64 years forming the largest age group. Weight was recorded in 14,964 reports (55.14%), including 10,426 reports for patients weighing <50 kg, 4,522 for those weighing 50–100 kg, and 16 for those weighing ≥100 kg (**Table 1**).

In CVARD, 4,020 reports had documented sex and 5,058 had age data. Adults aged 18–64 years (n = 2,787) formed the largest stated age group, followed by those aged 65–85 years (n = 2,211). Weight was recorded in 1,378 reports, including 85 weighing <50 kg, 1,205 weighing 50–100 kg, and 88 weighing >=100 kg. These are descriptive proportions among submitted reports with available data (**Table 1**).

Sex, age, and weight distributions differed across FAERS, JADER, and CVARD, but these findings are descriptive characteristics of submitted reports. Differences in reporting regulations, database structure, market use, coding, reporter composition, approval timing, follow-up procedures, and missingness preclude inference about geographic, biological, sex-specific, or low-body-weight susceptibility.

Annual report counts differed across databases and drugs. FAERS contained the largest number of capecitabine reports (**Figures 4A, B**), whereas 5-FU and tegafur were more frequently reported in JADER (**Figures 4C, D**), and capecitabine and 5-FU in CVARD (**Figures 4E, F**). The observed changes in annual counts were not interpreted as treatment-population event-frequency trends because exposure denominators and reporting practices varied over time.

**Figure 4:**
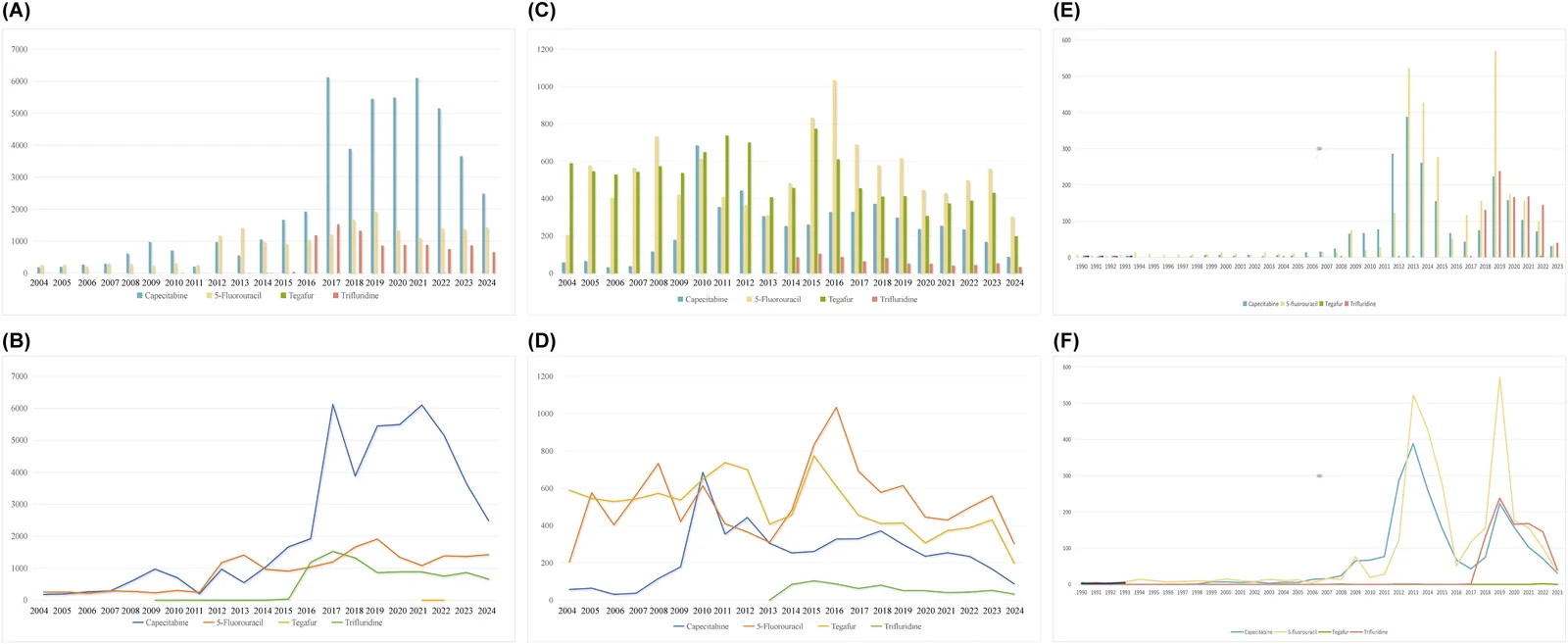
Annual counts of fluoropyrimidine-associated primary-suspect reports. **(A)** FAERS annual report-count bars, 2004–2024. **(B)** FAERS annual report-count lines. **(C)** JADER annual report-count bars, 2004–2024 Q2. **(D)** JADER annual report-count lines. **(E)** CVARD annual report-count bars, 1990–2023. **(F)** CVARD annual report-count lines. Annual counts describe reporting volume and not event frequency among treated populations.

Among FAERS reports with country information, the United States contributed the largest number, followed by France, Japan, Italy, and Canada (**Figure 5**). Country distributions reflect reporting pathways and database coverage and do not estimate country-specific event frequency or susceptibility.

**Figure 5:**
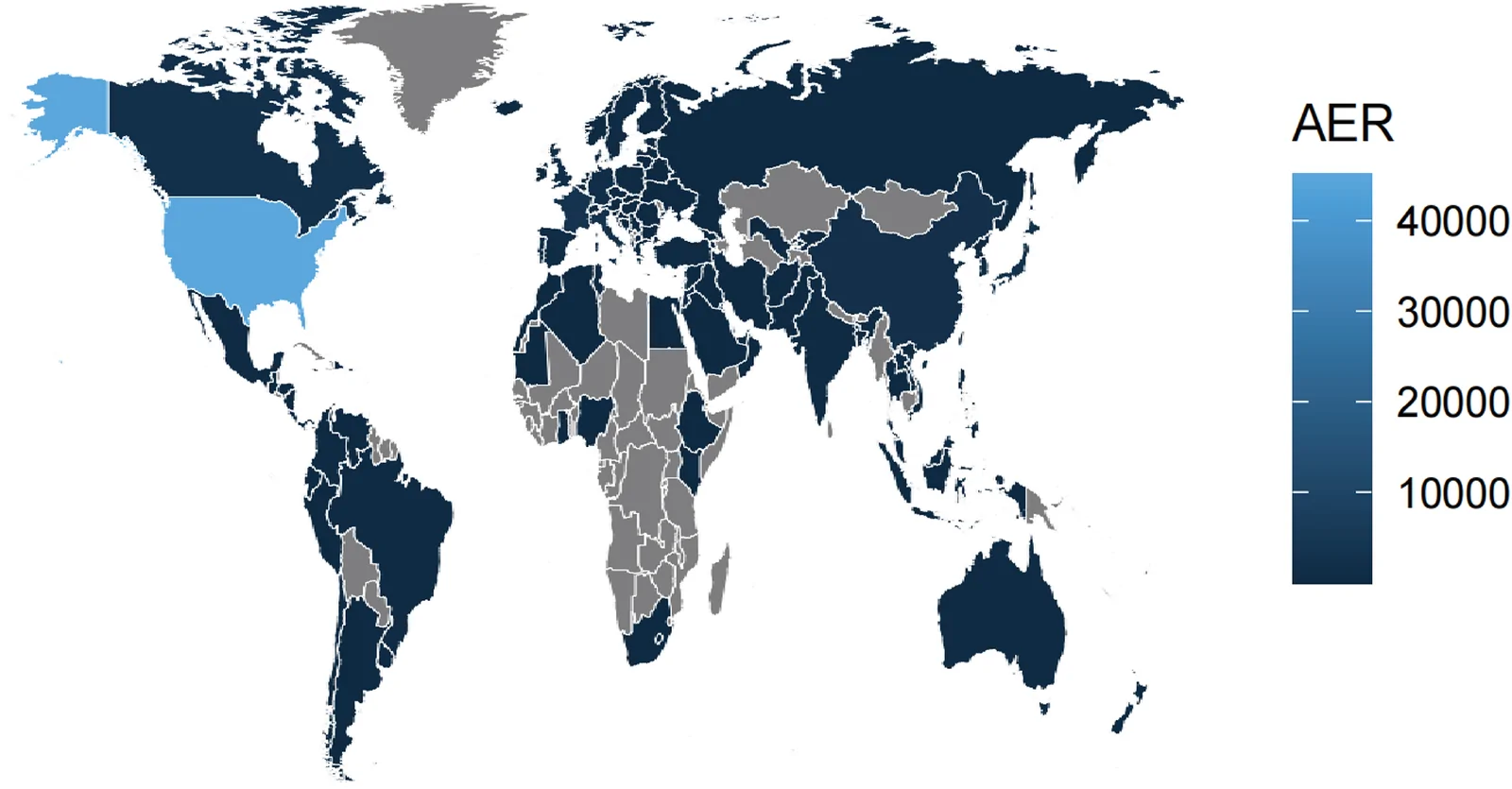
Country of origin of fluoropyrimidine-associated reports submitted to FAERS. Colors indicate numbers of submitted reports by country; they do not estimate country-specific event frequency or susceptibility.

### 3.2 Signal detection at the SOC level

All 27 MedDRA system organ classes (SOCs) were evaluated separately in FAERS, JADER, and CVARD (**Figures 6A–C**; **Tables 2**–**4**). In FAERS, gastrointestinal disorders (n = 41,125; ROR = 2.45), general disorders and administration-site conditions (n = 39,840; ROR = 1.04), and skin and subcutaneous-tissue disorders (n = 16,854; ROR = 1.45) were frequently reported (**Figure 6A**; **Table 2**). Among the eight most frequently reported FAERS SOCs, blood and lymphatic system disorders and gastrointestinal disorders showed the strongest reporting disproportionality (**Figure 6D**). These estimates do not quantify the probability of an event in treated patients.

**Figure 6:**
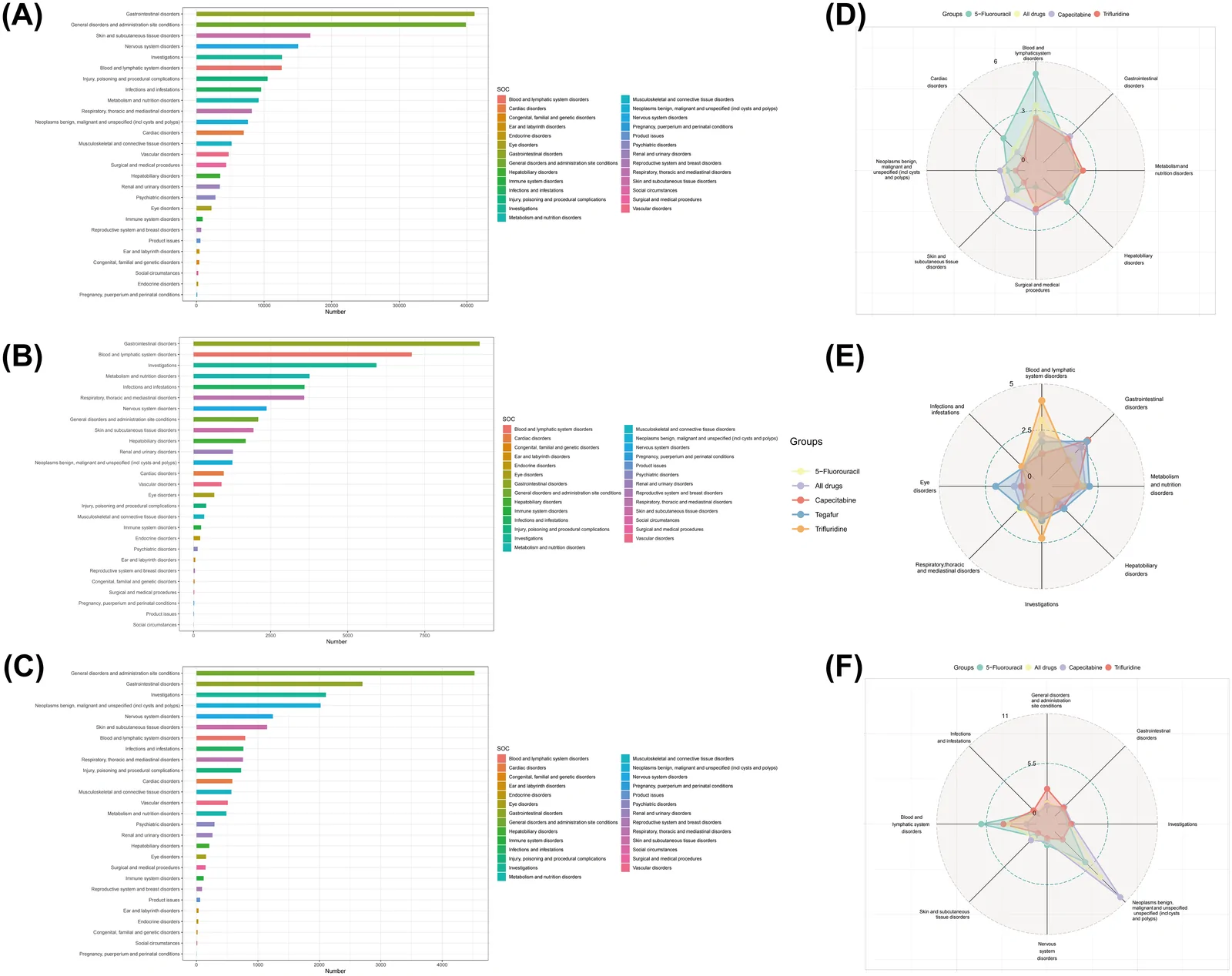
Disproportionality analyses at the system-organ-class level. **(A)** FAERS SOC report counts. **(B)** JADER SOC report counts. **(C)** CVARD SOC report counts. **(D–F)** ROR values for the eight most frequently reported SOCs in FAERS, JADER, and CVARD, respectively.

**Table 2:** Disproportionality estimates for fluoropyrimidine-associated reports at the system organ class level in FAERS.

SOC	Report count	ROR (95% CI)	PRR (χ^2^)	EBGM (EBGM05)	IC (IC025)
Gastrointestinal disorders	41125	2.45 ( 2.42 - 2.48 )	2.18 ( 28482.22 )	2.17 ( 2.15 )	1.12 ( 1.1 )
General disorders and administration site conditions	39840	1.04 ( 1.03 - 1.05 )	1.03 ( 45.09 )	1.03 ( 1.02 )	0.04 ( 0.03 )
Skin and subcutaneous tissue disorders	16854	1.45 ( 1.42 - 1.47 )	1.41 ( 2125.72 )	1.41 ( 1.39 )	0.49 ( 0.47 )
Nervous system disorders	15033	0.79 ( 0.77 - 0.8 )	0.8 ( 815.97 )	0.8 ( 0.79 )	-0.32 ( -0.34 )
Investigations	12642	0.92 ( 0.9 - 0.93 )	0.92 ( 92.62 )	0.92 ( 0.91 )	-0.12 ( -0.15 )
Blood and lymphatic system disorders	12600	3.5 ( 3.44 - 3.57 )	3.36 ( 20950.18 )	3.33 ( 3.28 )	1.73 ( 1.71 )
Injury, poisoning and procedural complications	10515	0.48 ( 0.47 - 0.49 )	0.5 ( 5760.16 )	0.5 ( 0.49 )	-0.99 ( -1.02 )
Infections and infestations	9567	0.81 ( 0.79 - 0.82 )	0.82 ( 421.02 )	0.82 ( 0.8 )	-0.29 ( -0.32 )
Metabolism and nutrition disorders	9193	1.98 ( 1.94 - 2.02 )	1.94 ( 4226.31 )	1.93 ( 1.9 )	0.95 ( 0.92 )
Respiratory, thoracic and mediastinal disorders	8169	0.76 ( 0.74 - 0.78 )	0.77 ( 586.1 )	0.77 ( 0.76 )	-0.38 ( -0.41 )
Neoplasms benign, malignant and unspecified (incl cysts and polyps)	7606	1.31 ( 1.28 - 1.34 )	1.3 ( 541.34 )	1.3 ( 1.27 )	0.38 ( 0.34 )
Cardiac disorders	7006	1.2 ( 1.18 - 1.23 )	1.2 ( 232.36 )	1.2 ( 1.17 )	0.26 ( 0.22 )
Musculoskeletal and connective tissue disorders	5187	0.43 ( 0.42 - 0.44 )	0.44 ( 3851.34 )	0.44 ( 0.43 )	-1.17 ( -1.22 )
Vascular disorders	4762	1 ( 0.97 - 1.02 )	1 ( 0.09 )	1 ( 0.97 )	-0.01 ( -0.05 )
Surgical and medical procedures	4390	1.47 ( 1.42 - 1.51 )	1.46 ( 636.48 )	1.46 ( 1.42 )	0.54 ( 0.5 )
Hepatobiliary disorders	3510	1.75 ( 1.69 - 1.81 )	1.73 ( 1094.12 )	1.73 ( 1.68 )	0.79 ( 0.74 )
Renal and urinary disorders	3455	0.84 ( 0.82 - 0.87 )	0.85 ( 97.2 )	0.85 ( 0.82 )	-0.24 ( -0.29 )
Psychiatric disorders	2818	0.21 ( 0.21 - 0.22 )	0.22 ( 8081.75 )	0.22 ( 0.22 )	-2.16 ( -2.21 )
Eye disorders	2214	0.49 ( 0.47 - 0.51 )	0.5 ( 1155.59 )	0.5 ( 0.48 )	-1.01 ( -1.07 )
Immune system disorders	907	0.36 ( 0.34 - 0.39 )	0.37 ( 1005.83 )	0.37 ( 0.35 )	-1.45 ( -1.54 )
Reproductive system and breast disorders	695	0.38 ( 0.35 - 0.41 )	0.38 ( 701.13 )	0.38 ( 0.36 )	-1.39 ( -1.5 )
Product issues	590	0.17 ( 0.15 - 0.18 )	0.17 ( 2478.05 )	0.17 ( 0.16 )	-2.57 ( -2.69 )
Ear and labyrinth disorders	434	0.45 ( 0.41 - 0.5 )	0.45 ( 286.45 )	0.45 ( 0.42 )	-1.14 ( -1.28 )
Congenital, familial and genetic disorders	399	0.59 ( 0.53 - 0.65 )	0.59 ( 114.21 )	0.59 ( 0.54 )	-0.76 ( -0.9 )
Social circumstances	274	0.28 ( 0.25 - 0.32 )	0.28 ( 499.26 )	0.28 ( 0.26 )	-1.82 ( -1.99 )
Endocrine disorders	252	0.44 ( 0.39 - 0.5 )	0.44 ( 175.42 )	0.45 ( 0.4 )	-1.17 ( -1.35 )
Pregnancy, puerperium and perinatal conditions	119	0.12 ( 0.1 - 0.15 )	0.12 ( 732.59 )	0.13 ( 0.11 )	-3 ( -3.26 )

**Table 3:** Disproportionality estimates for fluoropyrimidine-associated reports at the system organ class level in JADER.

SOC	Report count	ROR (95% CI)	PRR (χ^2^)	EBGM (EBGM05)	IC (IC025)
Gastrointestinal disorders	9279	2.96 ( 2.89 - 3.03 )	2.58 ( 9011.68 )	2.46 ( 2.4 )	1.3 ( -0.37 )
Blood and lymphatic system disorders	7078	2.57 ( 2.51 - 2.64 )	2.34 ( 5421.73 )	2.25 ( 2.19 )	1.17 ( -0.5 )
Investigations	5934	1.35 ( 1.31 - 1.39 )	1.31 ( 454.51 )	1.29 ( 1.26 )	0.37 ( -1.29 )
Metabolism and nutrition disorders	3762	1.93 ( 1.86 - 2 )	1.86 ( 1467.34 )	1.81 ( 1.75 )	0.86 ( -0.81 )
Infections and infestations	3599	0.92 ( 0.88 - 0.95 )	0.92 ( 25.52 )	0.92 ( 0.89 )	-0.11 ( -1.78 )
Respiratory, thoracic and mediastinal disorders	3592	1.03 ( 1 - 1.07 )	1.03 ( 3.39 )	1.03 ( 0.99 )	0.04 ( -1.62 )
Nervous system disorders	2370	0.49 ( 0.47 - 0.51 )	0.52 ( 1160 )	0.52 ( 0.5 )	-0.93 ( -2.6 )
General disorders and administration site conditions	2098	0.64 ( 0.62 - 0.67 )	0.66 ( 386.25 )	0.67 ( 0.64 )	-0.59 ( -2.25 )
Skin and subcutaneous tissue disorders	1946	0.64 ( 0.61 - 0.67 )	0.66 ( 367.67 )	0.66 ( 0.63 )	-0.59 ( -2.26 )
Hepatobiliary disorders	1691	0.86 ( 0.82 - 0.9 )	0.86 ( 37.46 )	0.87 ( 0.82 )	-0.21 ( -1.87 )
Renal and urinary disorders	1279	0.66 ( 0.63 - 0.7 )	0.67 ( 205.99 )	0.68 ( 0.64 )	-0.56 ( -2.22 )
Neoplasms benign, malignant and unspecified (incl cysts and polyps)	1263	0.66 ( 0.62 - 0.69 )	0.66 ( 218.31 )	0.67 ( 0.63 )	-0.57 ( -2.24 )
Cardiac disorders	979	0.48 ( 0.45 - 0.51 )	0.49 ( 544.58 )	0.49 ( 0.46 )	-1.02 ( -2.68 )
Vascular disorders	908	0.71 ( 0.66 - 0.76 )	0.71 ( 103.75 )	0.72 ( 0.67 )	-0.47 ( -2.14 )
Eye disorders	675	0.93 ( 0.86 - 1 )	0.93 ( 3.43 )	0.93 ( 0.86 )	-0.1 ( -1.77 )
Injury, poisoning and procedural complications	412	0.28 ( 0.26 - 0.31 )	0.29 ( 734.85 )	0.3 ( 0.27 )	-1.76 ( -3.42 )
Musculoskeletal and connective tissue disorders	346	0.26 ( 0.23 - 0.29 )	0.26 ( 732.9 )	0.27 ( 0.24 )	-1.9 ( -3.57 )
Immune system disorders	246	0.16 ( 0.14 - 0.18 )	0.16 ( 1081.05 )	0.17 ( 0.15 )	-2.58 ( -4.24 )
Endocrine disorders	218	0.27 ( 0.23 - 0.3 )	0.27 ( 434.92 )	0.28 ( 0.24 )	-1.86 ( -3.52 )
Psychiatric disorders	136	0.13 ( 0.11 - 0.16 )	0.13 ( 771.69 )	0.14 ( 0.12 )	-2.86 ( -4.52 )
Ear and labyrinth disorders	57	0.52 ( 0.4 - 0.68 )	0.52 ( 24.45 )	0.53 ( 0.41 )	-0.91 ( -2.58 )
Reproductive system and breast disorders	46	0.24 ( 0.18 - 0.32 )	0.24 ( 108.79 )	0.25 ( 0.19 )	-2.01 ( -3.68 )
Congenital, familial and genetic disorders	33	0.27 ( 0.19 - 0.37 )	0.27 ( 66.59 )	0.27 ( 0.19 )	-1.88 ( -3.55 )
Surgical and medical procedures	25	0.12 ( 0.08 - 0.18 )	0.12 ( 156.09 )	0.13 ( 0.09 )	-2.98 ( -4.65 )
Pregnancy, puerperium and perinatal conditions	22	0.11 ( 0.08 - 0.17 )	0.11 ( 150.72 )	0.12 ( 0.08 )	-3.09 ( -4.75 )
Product issues	16	0.59 ( 0.36 - 0.96 )	0.59 ( 4.54 )	0.6 ( 0.36 )	-0.75 ( -2.42 )
Social circumstances	7	0.28 ( 0.13 - 0.58 )	0.28 ( 13.17 )	0.28 ( 0.13 )	-1.82 ( -3.49 )

**Table 4:** Disproportionality estimates for fluoropyrimidine-associated reports at the system organ class level in CVARD.

SOC	Report count	ROR (95% CI)	PRR (χ^2^)	EBGM (EBGM05)	IC (IC025)
General disorders and administration site conditions	4526	1.09 ( 1.06 - 1.13 )	1.07 ( 26.88 )	1.07 ( 1.04 )	0.1 ( -1.57 )
Gastrointestinal disorders	2702	1.38 ( 1.32 - 1.43 )	1.33 ( 238.5 )	1.32 ( 1.27 )	0.4 ( -1.26 )
Investigations	2106	1.36 ( 1.3 - 1.42 )	1.32 ( 174.36 )	1.32 ( 1.26 )	0.4 ( -1.27 )
Neoplasms benign, malignant and unspecified (incl cysts and polyps)	2021	7.1 ( 6.78 - 7.44 )	6.5 ( 9193.01 )	6.29 ( 6 )	2.65 ( 0.99 )
Nervous system disorders	1244	0.81 ( 0.76 - 0.85 )	0.82 ( 54.53 )	0.82 ( 0.77 )	-0.29 ( -1.96 )
Skin and subcutaneous tissue disorders	1151	0.95 ( 0.89 - 1.01 )	0.95 ( 2.86 )	0.95 ( 0.9 )	-0.07 ( -1.74 )
Blood and lymphatic system disorders	793	3.13 ( 2.91 - 3.36 )	3.04 ( 1082.57 )	3.01 ( 2.8 )	1.59 ( -0.08 )
Infections and infestations	764	0.54 ( 0.51 - 0.59 )	0.56 ( 279.62 )	0.56 ( 0.52 )	-0.83 ( -2.5 )
Respiratory, thoracic and mediastinal disorders	755	0.62 ( 0.58 - 0.67 )	0.64 ( 163.95 )	0.64 ( 0.59 )	-0.65 ( -2.31 )
Injury, poisoning and procedural complications	728	0.5 ( 0.46 - 0.54 )	0.52 ( 349.91 )	0.52 ( 0.48 )	-0.95 ( -2.61 )
Cardiac disorders	584	1.5 ( 1.38 - 1.63 )	1.49 ( 94.14 )	1.48 ( 1.37 )	0.57 ( -1.1 )
Musculoskeletal and connective tissue disorders	568	0.39 ( 0.36 - 0.43 )	0.41 ( 520.74 )	0.41 ( 0.38 )	-1.29 ( -2.95 )
Vascular disorders	509	1.14 ( 1.05 - 1.25 )	1.14 ( 8.92 )	1.14 ( 1.04 )	0.19 ( -1.48 )
Metabolism and nutrition disorders	486	1.5 ( 1.37 - 1.64 )	1.49 ( 77.86 )	1.48 ( 1.35 )	0.57 ( -1.1 )
Psychiatric disorders	293	0.32 ( 0.28 - 0.36 )	0.33 ( 422.62 )	0.33 ( 0.29 )	-1.61 ( -3.27 )
Renal and urinary disorders	261	1.06 ( 0.94 - 1.2 )	1.06 ( 0.99 )	1.06 ( 0.94 )	0.09 ( -1.58 )
Hepatobiliary disorders	208	1.59 ( 1.39 - 1.83 )	1.59 ( 45.21 )	1.58 ( 1.38 )	0.66 ( -1 )
Eye disorders	155	0.45 ( 0.38 - 0.53 )	0.45 ( 103.9 )	0.45 ( 0.39 )	-1.14 ( -2.8 )
Surgical and medical procedures	148	0.61 ( 0.52 - 0.72 )	0.61 ( 36.63 )	0.61 ( 0.52 )	-0.7 ( -2.37 )
Immune system disorders	116	0.54 ( 0.45 - 0.65 )	0.55 ( 43.8 )	0.55 ( 0.46 )	-0.87 ( -2.53 )
Reproductive system and breast disorders	89	0.65 ( 0.52 - 0.8 )	0.65 ( 17.09 )	0.65 ( 0.53 )	-0.62 ( -2.29 )
Product issues	60	0.31 ( 0.24 - 0.4 )	0.31 ( 91.11 )	0.31 ( 0.24 )	-1.67 ( -3.34 )
Ear and labyrinth disorders	33	0.39 ( 0.28 - 0.55 )	0.39 ( 31.79 )	0.39 ( 0.28 )	-1.36 ( -3.02 )
Endocrine disorders	29	0.76 ( 0.53 - 1.1 )	0.76 ( 2.12 )	0.76 ( 0.53 )	-0.39 ( -2.05 )
Congenital, familial and genetic disorders	17	0.64 ( 0.4 - 1.04 )	0.64 ( 3.32 )	0.65 ( 0.4 )	-0.63 ( -2.3 )
Social circumstances	13	0.16 ( 0.09 - 0.27 )	0.16 ( 59.58 )	0.16 ( 0.09 )	-2.67 ( -4.34 )
Pregnancy, puerperium and perinatal conditions	3	0.05 ( 0.02 - 0.15 )	0.05 ( 57.68 )	0.05 ( 0.02 )	-4.39 ( -6.06 )

In JADER, the predominant SOCs were gastrointestinal disorders (n = 9,279; ROR = 2.96), blood and lymphatic system disorders (n = 7,078; ROR = 2.57), and investigations (n = 5,934; ROR = 1.35) (**Figure 6B**; **Table 3**). Among its eight highest-frequency SOCs, gastrointestinal disorders and blood and lymphatic system disorders demonstrated the strongest reporting disproportionality (**Figure 6E**).

In CVARD, general disorders and administration-site conditions (n = 4,526; ROR = 1.09), gastrointestinal disorders (n = 2,702; ROR = 1.38), and investigations (n = 2,106; ROR = 1.36) were the most frequently reported SOCs (**Figure 6C**; **Table 4**). Among CVARD’s eight highest-frequency SOCs, neoplasms benign, malignant and unspecified (including cysts and polyps) and blood and lymphatic system disorders showed the strongest reporting disproportionality (**Figure 6F**).

Gastrointestinal disorders showed substantial reporting frequency and disproportionality across the three databases. Because this SOC is clinically relevant to fluoropyrimidine therapy, it was selected for detailed analysis.

### 3.3 Signal detection at the PT level

**Tables 5**–**7** summarize the 50 most frequently reported preferred terms (PTs) across all system organ classes in FAERS, JADER, and CVARD, respectively. Gastrointestinal-specific report counts, reporting proportions, disproportionality patterns, and reported case-fatality proportions are presented separately in **Figures 7** and **8**.

**Table 5:** The 50 most frequently reported preferred terms across all system organ classes with corresponding disproportionality estimates in FAERS.

PT	Report count	ROR (95% CI)	PRR (χ^2^)	EBGM (EBGM05)	IC (IC025)
Diarrhoea	9759	4.4 ( 4.32 - 4.5 )	4.25 ( 24131.75 )	4.2 ( 4.13 )	2.07 ( 2.04 )
Death	7360	2.43 ( 2.37 - 2.48 )	2.38 ( 5905.23 )	2.36 ( 2.32 )	1.24 ( 1.21 )
Nausea	6985	2.5 ( 2.44 - 2.56 )	2.45 ( 6006.56 )	2.43 ( 2.39 )	1.28 ( 1.25 )
Fatigue	5320	1.91 ( 1.86 - 1.96 )	1.89 ( 2233.98 )	1.88 ( 1.84 )	0.91 ( 0.87 )
Vomiting	4705	2.83 ( 2.75 - 2.92 )	2.8 ( 5405.92 )	2.78 ( 2.71 )	1.47 ( 1.43 )
Disease progression	4446	11.01 ( 10.68 - 11.35 )	10.81 ( 37983.19 )	10.4 ( 10.14 )	3.38 ( 3.33 )
Palmar-plantar erythrodysaesthesia syndrome	4343	64.56 ( 62.43 - 66.77 )	63.31 ( 211803.97 )	50.53 ( 49.13 )	5.66 ( 5.61 )
Neutropenia	2832	5.88 ( 5.66 - 6.1 )	5.81 ( 11049.16 )	5.7 ( 5.53 )	2.51 ( 2.46 )
Off label use	2824	0.93 ( 0.9 - 0.97 )	0.94 ( 12.75 )	0.94 ( 0.91 )	-0.1 ( -0.15 )
Asthenia	2597	1.9 ( 1.83 - 1.97 )	1.89 ( 1081.19 )	1.88 ( 1.82 )	0.91 ( 0.85 )
Decreased appetite	2465	3 ( 2.89 - 3.13 )	2.98 ( 3221.47 )	2.96 ( 2.86 )	1.56 ( 1.51 )
Dehydration	2304	4.79 ( 4.59 - 4.99 )	4.75 ( 6698.97 )	4.68 ( 4.52 )	2.23 ( 2.16 )
Drug ineffective	2103	0.43 ( 0.41 - 0.45 )	0.44 ( 1566.14 )	0.44 ( 0.42 )	-1.19 ( -1.26 )
Neuropathy peripheral	1943	5.98 ( 5.72 - 6.26 )	5.94 ( 7798.56 )	5.82 ( 5.6 )	2.54 ( 2.47 )
Thrombocytopenia	1863	4.68 ( 4.47 - 4.9 )	4.65 ( 5246.75 )	4.58 ( 4.41 )	2.2 ( 2.13 )
Anaemia	1821	2.6 ( 2.48 - 2.72 )	2.58 ( 1754.5 )	2.57 ( 2.47 )	1.36 ( 1.29 )
Abdominal pain	1815	2.16 ( 2.06 - 2.26 )	2.15 ( 1114.54 )	2.14 ( 2.06 )	1.1 ( 1.03 )
Pyrexia	1756	1.38 ( 1.31 - 1.44 )	1.37 ( 177.69 )	1.37 ( 1.32 )	0.45 ( 0.39 )
Stomatitis	1675	7.85 ( 7.47 - 8.24 )	7.8 ( 9628.03 )	7.59 ( 7.28 )	2.92 ( 2.85 )
Mucosal inflammation	1631	18.64 ( 17.72 - 19.6 )	18.51 ( 25126.77 )	17.28 ( 16.56 )	4.11 ( 4.04 )
Febrile neutropenia	1556	6.69 ( 6.36 - 7.04 )	6.65 ( 7278.34 )	6.5 ( 6.23 )	2.7 ( 2.63 )
Dyspnoea	1489	0.72 ( 0.68 - 0.75 )	0.72 ( 166.89 )	0.72 ( 0.69 )	-0.48 ( -0.55 )
Pain	1416	0.62 ( 0.58 - 0.65 )	0.62 ( 337.67 )	0.62 ( 0.59 )	-0.69 ( -0.77 )
Rash	1414	0.91 ( 0.87 - 0.96 )	0.91 ( 11.94 )	0.91 ( 0.87 )	-0.13 ( -0.21 )
Malignant neoplasm progression	1407	3.98 ( 3.77 - 4.19 )	3.96 ( 3067.35 )	3.91 ( 3.74 )	1.97 ( 1.89 )
Constipation	1368	1.8 ( 1.7 - 1.9 )	1.79 ( 477.15 )	1.79 ( 1.71 )	0.84 ( 0.76 )
Weight decreased	1298	1.27 ( 1.2 - 1.34 )	1.27 ( 73.99 )	1.27 ( 1.21 )	0.34 ( 0.26 )
Pain in extremity	1204	1.09 ( 1.03 - 1.15 )	1.09 ( 8.91 )	1.09 ( 1.04 )	0.12 ( 0.04 )
Paraesthesia	1124	1.92 ( 1.81 - 2.04 )	1.92 ( 492.07 )	1.91 ( 1.82 )	0.93 ( 0.85 )
Erythema	1121	1.5 ( 1.41 - 1.59 )	1.5 ( 184.34 )	1.49 ( 1.42 )	0.58 ( 0.49 )
White blood cell count decreased	1095	2.76 ( 2.6 - 2.93 )	2.75 ( 1211.56 )	2.73 ( 2.6 )	1.45 ( 1.36 )
Malaise	1094	0.67 ( 0.63 - 0.71 )	0.67 ( 181.35 )	0.67 ( 0.64 )	-0.58 ( -0.67 )
Dizziness	1083	0.59 ( 0.56 - 0.63 )	0.59 ( 305.29 )	0.59 ( 0.56 )	-0.75 ( -0.84 )
Leukopenia	1081	6.05 ( 5.69 - 6.43 )	6.02 ( 4425.17 )	5.9 ( 5.61 )	2.56 ( 2.47 )
Skin exfoliation	1076	3.7 ( 3.48 - 3.93 )	3.69 ( 2078.42 )	3.65 ( 3.47 )	1.87 ( 1.78 )
Therapy interrupted	961	4.73 ( 4.44 - 5.05 )	4.72 ( 2765.06 )	4.65 ( 4.41 )	2.22 ( 2.12 )
Pneumonia	960	0.82 ( 0.77 - 0.88 )	0.82 ( 36.88 )	0.82 ( 0.78 )	-0.28 ( -0.37 )
Abdominal pain upper	960	1.3 ( 1.22 - 1.38 )	1.29 ( 64.03 )	1.29 ( 1.23 )	0.37 ( 0.28 )
Headache	956	0.41 ( 0.39 - 0.44 )	0.41 ( 796.69 )	0.42 ( 0.39 )	-1.27 ( -1.36 )
Chest pain	922	1.34 ( 1.26 - 1.43 )	1.34 ( 78.66 )	1.34 ( 1.27 )	0.42 ( 0.32 )
Dry skin	920	2.01 ( 1.88 - 2.14 )	2 ( 458.81 )	1.99 ( 1.89 )	1 ( 0.9 )
Sepsis	888	2.18 ( 2.04 - 2.33 )	2.17 ( 557.73 )	2.16 ( 2.05 )	1.11 ( 1.01 )
Hypoaesthesia	853	1.54 ( 1.44 - 1.65 )	1.54 ( 158.87 )	1.53 ( 1.45 )	0.62 ( 0.52 )
Platelet count decreased	830	2.13 ( 1.98 - 2.28 )	2.12 ( 488.56 )	2.11 ( 1.99 )	1.08 ( 0.98 )
Skin discolouration	825	4.98 ( 4.65 - 5.34 )	4.97 ( 2564.94 )	4.89 ( 4.62 )	2.29 ( 2.19 )
Pulmonary embolism	824	2.32 ( 2.17 - 2.49 )	2.32 ( 613.09 )	2.31 ( 2.18 )	1.21 ( 1.1 )
Therapy cessation	817	4.5 ( 4.2 - 4.82 )	4.48 ( 2174.42 )	4.42 ( 4.17 )	2.14 ( 2.04 )
Toxicity to various agents	814	1.36 ( 1.27 - 1.46 )	1.36 ( 78.54 )	1.36 ( 1.28 )	0.44 ( 0.34 )
Hospitalisation	812	1.53 ( 1.43 - 1.64 )	1.53 ( 147.13 )	1.52 ( 1.44 )	0.61 ( 0.51 )
Alopecia	789	1.08 ( 1.01 - 1.16 )	1.08 ( 4.97 )	1.08 ( 1.02 )	0.11 ( 0.01 )

**Table 6:** The 50 most frequently reported preferred terms across all system organ classes with corresponding disproportionality estimates in JADER.

PT	Report count	ROR (95% CI)	PRR (χ^2^)	EBGM (EBGM05)	IC (IC025)
Diarrhoea	2766	8.9 ( 8.53 - 9.29 )	8.45 ( 14433.48 )	6.87 ( 6.58 )	2.78 ( 1.11 )
Interstitial lung disease	2270	1.83 ( 1.76 - 1.92 )	1.8 ( 777.63 )	1.75 ( 1.68 )	0.81 ( -0.86 )
Neutrophil count decreased	1954	3.47 ( 3.31 - 3.64 )	3.37 ( 2983.52 )	3.14 ( 3 )	1.65 ( -0.01 )
Decreased appetite	1836	6.63 ( 6.3 - 6.98 )	6.42 ( 7023.66 )	5.5 ( 5.23 )	2.46 ( 0.79 )
Febrile neutropenia	1764	4.87 ( 4.63 - 5.12 )	4.73 ( 4543.09 )	4.24 ( 4.03 )	2.08 ( 0.42 )
Neutropenia	1702	4.17 ( 3.96 - 4.39 )	4.05 ( 3502.8 )	3.71 ( 3.52 )	1.89 ( 0.22 )
White blood cell count decreased	1142	2.52 ( 2.37 - 2.67 )	2.48 ( 943.83 )	2.37 ( 2.23 )	1.25 ( -0.42 )
Platelet count decreased	1131	1.62 ( 1.52 - 1.72 )	1.6 ( 249.1 )	1.58 ( 1.48 )	0.66 ( -1.01 )
Stomatitis	1029	11.49 ( 10.69 - 12.34 )	11.26 ( 7102.25 )	8.56 ( 7.96 )	3.1 ( 1.43 )
Nausea	897	3.16 ( 2.95 - 3.39 )	3.12 ( 1183.19 )	2.93 ( 2.73 )	1.55 ( -0.12 )
Palmar-plantar erythrodysaesthesia syndrome	746	14.5 ( 13.29 - 15.82 )	14.29 ( 6349.53 )	10.14 ( 9.29 )	3.34 ( 1.67 )
Leukopenia	726	5 ( 4.62 - 5.41 )	4.94 ( 1978.69 )	4.41 ( 4.07 )	2.14 ( 0.47 )
Vomiting	701	2.98 ( 2.76 - 3.23 )	2.96 ( 833.44 )	2.79 ( 2.58 )	1.48 ( -0.19 )
Pneumonia	610	1.02 ( 0.94 - 1.11 )	1.02 ( 0.25 )	1.02 ( 0.94 )	0.03 ( -1.64 )
Myelosuppression	573	2.48 ( 2.28 - 2.7 )	2.46 ( 463.71 )	2.36 ( 2.16 )	1.24 ( -0.43 )
Hyperammonaemia	571	38.01 ( 33.65 - 42.94 )	37.57 ( 9268.44 )	17.66 ( 15.64 )	4.14 ( 2.47 )
Anaemia	551	1.13 ( 1.03 - 1.23 )	1.13 ( 7.53 )	1.12 ( 1.03 )	0.17 ( -1.5 )
Thrombocytopenia	545	2.29 ( 2.1 - 2.5 )	2.28 ( 365.99 )	2.19 ( 2.01 )	1.13 ( -0.53 )
Disseminated intravascular coagulation	502	3.89 ( 3.54 - 4.27 )	3.86 ( 950.07 )	3.55 ( 3.23 )	1.83 ( 0.16 )
Hepatic function abnormal	496	0.74 ( 0.67 - 0.81 )	0.74 ( 44.73 )	0.75 ( 0.68 )	-0.42 ( -2.09 )
Pyrexia	483	0.7 ( 0.64 - 0.76 )	0.7 ( 62.05 )	0.71 ( 0.64 )	-0.5 ( -2.17 )
Malaise	439	1.7 ( 1.54 - 1.87 )	1.69 ( 119.19 )	1.66 ( 1.51 )	0.73 ( -0.94 )
Dehydration	427	4.34 ( 3.92 - 4.81 )	4.31 ( 957.72 )	3.91 ( 3.54 )	1.97 ( 0.3 )
Enterocolitis	419	6.58 ( 5.92 - 7.31 )	6.53 ( 1627.56 )	5.58 ( 5.02 )	2.48 ( 0.81 )
Renal impairment	387	0.75 ( 0.68 - 0.83 )	0.75 ( 31.83 )	0.76 ( 0.68 )	-0.4 ( -2.07 )
Haemoglobin decreased	386	2.4 ( 2.17 - 2.67 )	2.39 ( 291.8 )	2.29 ( 2.07 )	1.2 ( -0.47 )
Sepsis	369	1.88 ( 1.7 - 2.09 )	1.88 ( 143.3 )	1.83 ( 1.64 )	0.87 ( -0.8 )
Neuropathy peripheral	352	2.32 ( 2.08 - 2.58 )	2.31 ( 243.88 )	2.22 ( 1.99 )	1.15 ( -0.52 )
Pancytopenia	336	1.43 ( 1.28 - 1.6 )	1.43 ( 41.17 )	1.41 ( 1.26 )	0.49 ( -1.17 )
Acute kidney injury	330	1.02 ( 0.91 - 1.13 )	1.02 ( 0.08 )	1.01 ( 0.91 )	0.02 ( -1.64 )
Dacryostenosis acquired	261	73.04 ( 58.56 - 91.09 )	72.65 ( 5573.44 )	22.65 ( 18.16 )	4.5 ( 2.83 )
Altered state of consciousness	260	1.06 ( 0.93 - 1.19 )	1.05 ( 0.72 )	1.05 ( 0.93 )	0.07 ( -1.59 )
Rash	257	0.59 ( 0.52 - 0.67 )	0.6 ( 69.76 )	0.6 ( 0.53 )	-0.73 ( -2.4 )
Cerebral infarction	248	0.76 ( 0.67 - 0.86 )	0.76 ( 18.99 )	0.76 ( 0.67 )	-0.39 ( -2.06 )
Fatigue	243	5.17 ( 4.51 - 5.92 )	5.15 ( 699.1 )	4.57 ( 3.99 )	2.19 ( 0.52 )
Leukoencephalopathy	227	11.26 ( 9.67 - 13.1 )	11.21 ( 1556.88 )	8.53 ( 7.33 )	3.09 ( 1.42 )
Liver disorder	213	0.54 ( 0.47 - 0.62 )	0.54 ( 81.44 )	0.55 ( 0.48 )	-0.86 ( -2.53 )
Septic shock	201	2.41 ( 2.09 - 2.78 )	2.4 ( 153.22 )	2.3 ( 1.99 )	1.2 ( -0.46 )
Alanine aminotransferase increased	193	1.5 ( 1.3 - 1.73 )	1.5 ( 30.44 )	1.47 ( 1.28 )	0.56 ( -1.11 )
Cardiac failure	192	0.65 ( 0.56 - 0.75 )	0.65 ( 35.15 )	0.66 ( 0.57 )	-0.6 ( -2.27 )
Death	181	0.55 ( 0.47 - 0.63 )	0.55 ( 66.33 )	0.56 ( 0.48 )	-0.84 ( -2.51 )
Abdominal pain	178	2.33 ( 2 - 2.71 )	2.32 ( 124.85 )	2.23 ( 1.91 )	1.16 ( -0.51 )
Gastrointestinal perforation	173	3.25 ( 2.78 - 3.8 )	3.24 ( 243.02 )	3.03 ( 2.59 )	1.6 ( -0.07 )
Pulmonary embolism	172	1.66 ( 1.43 - 1.94 )	1.66 ( 42.96 )	1.63 ( 1.4 )	0.7 ( -0.96 )
Ileus	170	2.19 ( 1.88 - 2.56 )	2.19 ( 102.89 )	2.11 ( 1.81 )	1.08 ( -0.59 )
Aspartate aminotransferase increased	169	1.4 ( 1.2 - 1.64 )	1.4 ( 18.62 )	1.38 ( 1.19 )	0.47 ( -1.2 )
Stevens-Johnson syndrome	147	0.69 ( 0.59 - 0.82 )	0.69 ( 19.42 )	0.7 ( 0.6 )	-0.51 ( -2.18 )
Peritonitis	147	2.14 ( 1.81 - 2.53 )	2.13 ( 82.93 )	2.06 ( 1.74 )	1.04 ( -0.62 )
Malignant neoplasm progression	144	0.7 ( 0.6 - 0.83 )	0.7 ( 17.58 )	0.71 ( 0.6 )	-0.49 ( -2.16 )
Pneumonitis	143	1.22 ( 1.04 - 1.45 )	1.22 ( 5.66 )	1.22 ( 1.03 )	0.28 ( -1.38 )

**Table 7:** The 50 most frequently reported preferred terms across all system organ classes with corresponding disproportionality estimates in CVARD.

PT	Report count	ROR (95% CI)	PRR (χ^2^)	EBGM (EBGM05)	IC (IC025)
Disease progression	1271	61.22 ( 57.35 - 65.35 )	57.46 ( 52525.88 )	43 ( 40.28 )	5.43 ( 3.76 )
Diarrhoea	615	2.82 ( 2.6 - 3.06 )	2.77 ( 690.15 )	2.74 ( 2.53 )	1.45 ( -0.21 )
Malignant neoplasm progression	597	18.29 ( 16.79 - 19.92 )	17.78 ( 8560.53 )	16.17 ( 14.84 )	4.01 ( 2.35 )
Death	576	2.69 ( 2.48 - 2.93 )	2.64 ( 585.81 )	2.62 ( 2.41 )	1.39 ( -0.28 )
Nausea	400	1.37 ( 1.24 - 1.51 )	1.36 ( 38.64 )	1.36 ( 1.23 )	0.44 ( -1.22 )
Fatigue	397	1.41 ( 1.28 - 1.56 )	1.4 ( 45.96 )	1.4 ( 1.27 )	0.48 ( -1.18 )
Neoplasm progression	342	31.56 ( 28.1 - 35.45 )	31.05 ( 8390.63 )	26.33 ( 23.44 )	4.72 ( 3.05 )
Neutropenia	285	6.91 ( 6.14 - 7.79 )	6.83 ( 1365.34 )	6.6 ( 5.86 )	2.72 ( 1.06 )
Vomiting	270	1.6 ( 1.42 - 1.8 )	1.59 ( 59.34 )	1.59 ( 1.41 )	0.67 ( -1 )
Febrile neutropenia	257	12.5 ( 11 - 14.2 )	12.35 ( 2499.03 )	11.57 ( 10.18 )	3.53 ( 1.87 )
Neuropathy peripheral	252	16.48 ( 14.47 - 18.77 )	16.29 ( 3296.68 )	14.93 ( 13.11 )	3.9 ( 2.23 )
Asthenia	235	1.75 ( 1.54 - 2 )	1.74 ( 74.4 )	1.74 ( 1.53 )	0.8 ( -0.87 )
Drug ineffective	235	0.21 ( 0.18 - 0.24 )	0.22 ( 688.64 )	0.22 ( 0.19 )	-2.18 ( -3.85 )
Palmar-plantar erythrodysaesthesia syndrome	216	119.43 ( 100.27 - 142.25 )	118.17 ( 14693.06 )	69.59 ( 58.43 )	6.12 ( 4.45 )
Pyrexia	186	1.33 ( 1.15 - 1.54 )	1.33 ( 15.35 )	1.33 ( 1.15 )	0.41 ( -1.26 )
Breast cancer	179	12.15 ( 10.43 - 14.15 )	12.05 ( 1692.73 )	11.3 ( 9.71 )	3.5 ( 1.83 )
Decreased appetite	171	2.27 ( 1.95 - 2.64 )	2.26 ( 118.71 )	2.24 ( 1.93 )	1.16 ( -0.5 )
Colorectal cancer	165	125.59 ( 102.61 - 153.72 )	124.58 ( 11582.1 )	71.75 ( 58.62 )	6.16 ( 4.49 )
Pain	164	0.73 ( 0.62 - 0.85 )	0.73 ( 16.48 )	0.73 ( 0.63 )	-0.45 ( -2.12 )
Dyspnoea	152	0.62 ( 0.53 - 0.72 )	0.62 ( 35.77 )	0.62 ( 0.53 )	-0.69 ( -2.35 )
Toxicity to various agents	149	6.51 ( 5.52 - 7.67 )	6.47 ( 663.59 )	6.26 ( 5.31 )	2.65 ( 0.98 )
Abdominal pain	133	0.91 ( 0.77 - 1.08 )	0.91 ( 1.07 )	0.91 ( 0.77 )	-0.13 ( -1.79 )
Weight decreased	133	1.19 ( 1 - 1.41 )	1.19 ( 3.86 )	1.18 ( 1 )	0.24 ( -1.42 )
Rash	131	0.76 ( 0.64 - 0.9 )	0.76 ( 10.12 )	0.76 ( 0.64 )	-0.4 ( -2.06 )
Breast cancer metastatic	127	42.64 ( 35.08 - 51.84 )	42.38 ( 4092.96 )	34 ( 27.97 )	5.09 ( 3.42 )
Mucosal inflammation	120	29.57 ( 24.34 - 35.92 )	29.4 ( 2799.85 )	25.15 ( 20.7 )	4.65 ( 2.98 )
Dehydration	119	3.57 ( 2.97 - 4.28 )	3.55 ( 214.22 )	3.5 ( 2.92 )	1.81 ( 0.14 )
Malaise	118	0.76 ( 0.63 - 0.91 )	0.76 ( 9.22 )	0.76 ( 0.63 )	-0.4 ( -2.07 )
Stomatitis	106	6.99 ( 5.75 - 8.49 )	6.96 ( 519.54 )	6.72 ( 5.53 )	2.75 ( 1.08 )
Ejection fraction decreased	106	25.21 ( 20.55 - 30.94 )	25.09 ( 2131.68 )	21.94 ( 17.88 )	4.46 ( 2.79 )
Erythema	105	1.14 ( 0.94 - 1.38 )	1.14 ( 1.71 )	1.14 ( 0.94 )	0.18 ( -1.48 )
Chest pain	103	1.33 ( 1.09 - 1.61 )	1.33 ( 8.23 )	1.32 ( 1.09 )	0.4 ( -1.26 )
White blood cell count decreased	102	3.51 ( 2.89 - 4.28 )	3.5 ( 178.85 )	3.45 ( 2.83 )	1.79 ( 0.12 )
Dizziness	100	0.54 ( 0.44 - 0.65 )	0.54 ( 39.93 )	0.54 ( 0.44 )	-0.89 ( -2.56 )
Platelet count decreased	99	4.04 ( 3.3 - 4.93 )	4.02 ( 219.62 )	3.95 ( 3.23 )	1.98 ( 0.31 )
Headache	96	0.42 ( 0.34 - 0.51 )	0.42 ( 77.77 )	0.42 ( 0.34 )	-1.25 ( -2.91 )
Haemoglobin decreased	95	1.59 ( 1.3 - 1.95 )	1.59 ( 20.64 )	1.58 ( 1.29 )	0.66 ( -1 )
Pain in extremity	95	0.76 ( 0.62 - 0.93 )	0.76 ( 7.01 )	0.76 ( 0.62 )	-0.39 ( -2.05 )
Nasopharyngitis	93	0.96 ( 0.78 - 1.18 )	0.96 ( 0.15 )	0.96 ( 0.78 )	-0.06 ( -1.72 )
Metastases to liver	91	17.81 ( 14.35 - 22.12 )	17.74 ( 1299.6 )	16.13 ( 12.99 )	4.01 ( 2.34 )
Constipation	90	1.54 ( 1.25 - 1.89 )	1.54 ( 16.68 )	1.53 ( 1.24 )	0.61 ( -1.05 )
Back pain	89	0.93 ( 0.76 - 1.15 )	0.93 ( 0.42 )	0.93 ( 0.76 )	-0.1 ( -1.77 )
Hypoaesthesia	87	1.36 ( 1.1 - 1.68 )	1.36 ( 8.11 )	1.35 ( 1.1 )	0.44 ( -1.23 )
Peripheral swelling	87	1.11 ( 0.9 - 1.37 )	1.11 ( 0.92 )	1.11 ( 0.9 )	0.15 ( -1.52 )
Off label use	87	0.3 ( 0.24 - 0.37 )	0.3 ( 139.71 )	0.31 ( 0.25 )	-1.71 ( -3.37 )
Infection	84	1.41 ( 1.14 - 1.75 )	1.41 ( 9.91 )	1.41 ( 1.13 )	0.49 ( -1.18 )
Pneumonia	83	0.43 ( 0.34 - 0.53 )	0.43 ( 63.65 )	0.43 ( 0.35 )	-1.22 ( -2.88 )
Paraesthesia	82	1.51 ( 1.21 - 1.87 )	1.5 ( 13.74 )	1.5 ( 1.21 )	0.58 ( -1.08 )
Pulmonary embolism	76	2.76 ( 2.2 - 3.46 )	2.75 ( 83.4 )	2.72 ( 2.17 )	1.44 ( -0.22 )
Cough	74	0.57 ( 0.45 - 0.71 )	0.57 ( 24.2 )	0.57 ( 0.45 )	-0.81 ( -2.48 )

**Figure 7:**
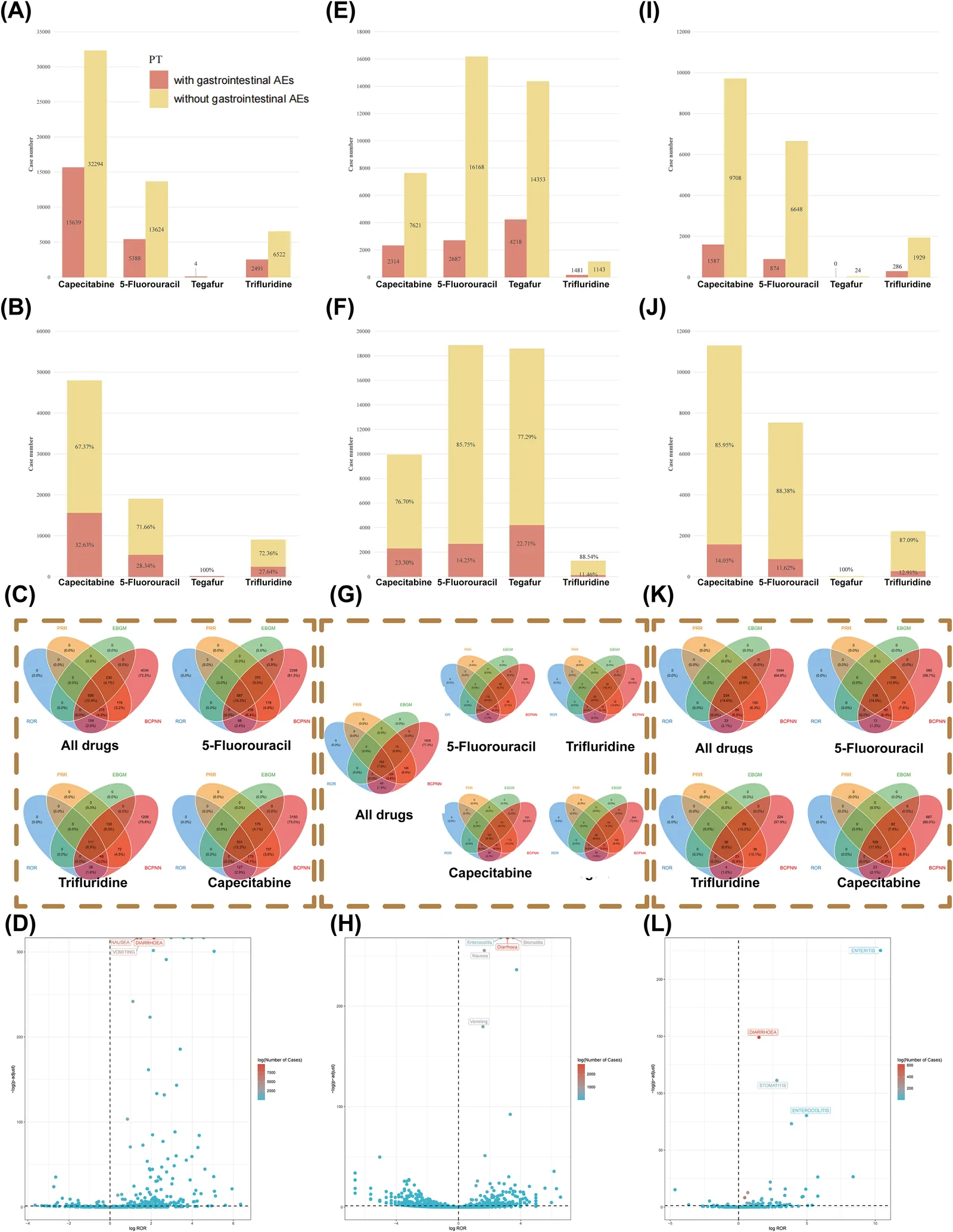
Disproportionality analyses at the preferred-term level. **(A–D)** FAERS gastrointestinal report counts, proportions among submitted reports, concordance across four correlated signal-detection methods, and ROR volcano plot. **(E–H)** corresponding JADER analyses. **(I–L)** corresponding CVARD analyses. Drug-event relations are described as reporting associations, not causation.

**Figure 8:**
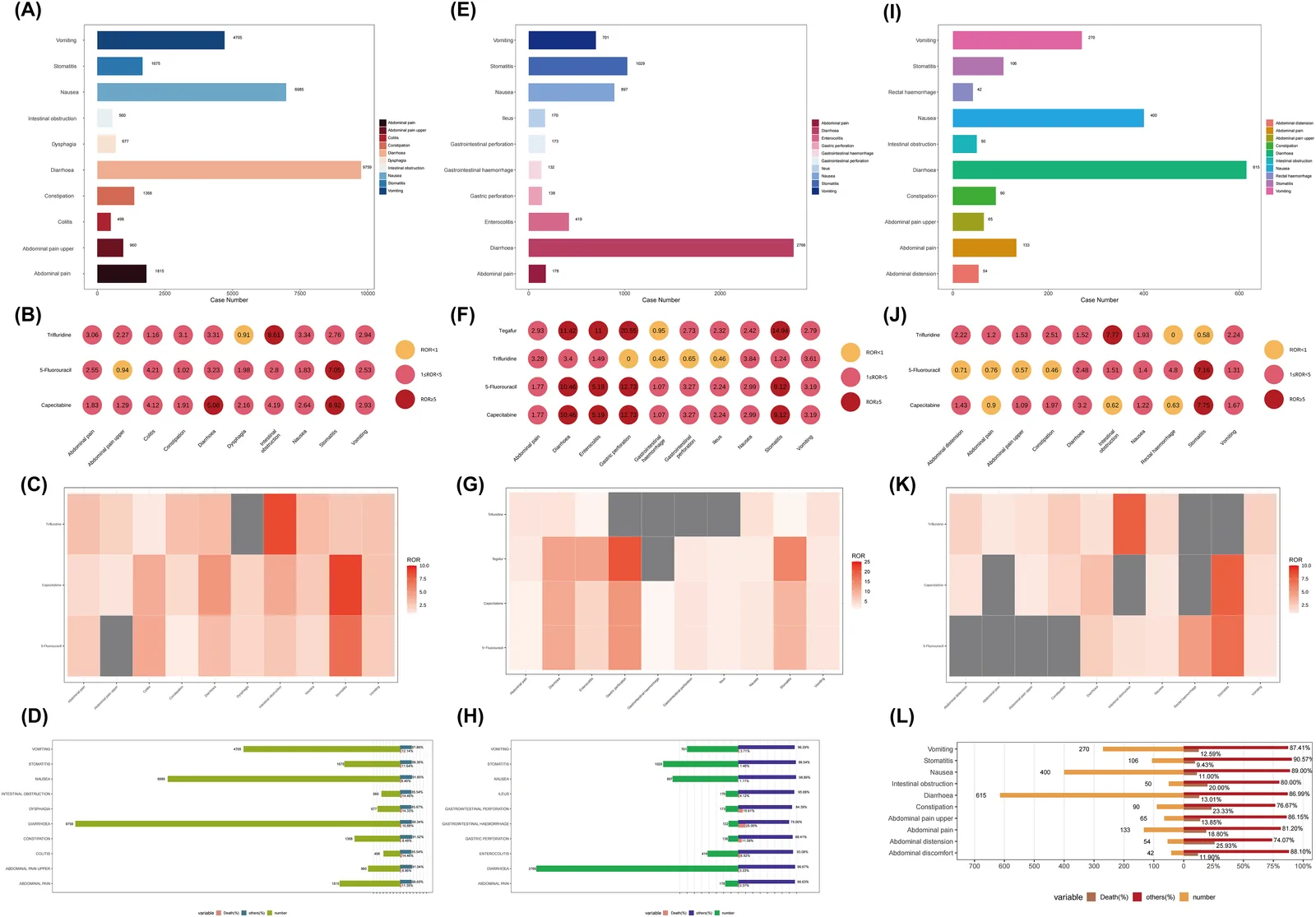
Reporting patterns for the ten most frequently reported gastrointestinal preferred terms. **(A–D)** FAERS counts, ROR heatmaps, and reported case-fatality proportions. **(E–H)** corresponding JADER analyses. **(I–L)** corresponding CVARD analyses. Case-fatality proportions summarize the analysis and do not estimate fatality among treated patients.

In FAERS, gastrointestinal-disorder reports accounted for 15,639 capecitabine reports (32.63%), 5,388 5-FU reports (28.34%), 2,491 trifluridine reports (27.64%), and 4 tegafur reports (100%) (**Figures 7A, B**). These are proportions among submitted reports and do not represent event frequency among treated populations. Across the four correlated methods, 690 PTs met all prespecified criteria (**Figure 7C**), and the ROR volcano plot is shown in **Figure 7D**. The 50 most frequently reported PTs across all SOCs are listed in **Table 5**. In the gastrointestinal-focused analysis, diarrhea, nausea, and vomiting were frequently reported (**Figure 8A**); capecitabine showed marked reporting disproportionality for stomatitis (ROR = 8.92; **Figures 8B, C**); and intestinal obstruction/colitis had a reported case-fatality proportion of 14.46% (**Figure 8D**).

In JADER, gastrointestinal-disorder reports accounted for 2,314 capecitabine reports (23.30%), 2,687 5-FU reports (14.25%), 1,481 trifluridine reports (11.46%), and 4,218 tegafur reports (22.71%) (**Figures 7E, F**). These are reporting proportions among submitted reports. Across the four correlated methods, 162 PTs met all prespecified criteria (**Figure 7G**), and the ROR volcano plot is shown in **Figure 7H**. The 50 most frequently reported PTs across all SOCs are listed in **Table 6**. In the gastrointestinal-focused analysis, tegafur showed marked reporting disproportionality for gastric perforation (ROR = 20.55; **Figures 8E–G**), and gastrointestinal hemorrhage had a reported case-fatality proportion of 25.00% (**Figure 8H**).

In CVARD, gastrointestinal-disorder reports accounted for 1,587 capecitabine reports (14.05%), 874 5-FU reports (11.62%), 286 trifluridine reports (12.91%), and no tegafur reports (**Figures 7I, J**). These are reporting proportions among submitted reports. Across the four correlated methods, 234 PTs met all prespecified criteria (**Figure 7K**), and the ROR volcano plot is shown in **Figure 7L**. The 50 most frequently reported PTs across all SOCs are listed in **Table 7**. In the gastrointestinal-focused analysis, trifluridine showed marked reporting disproportionality for intestinal obstruction (ROR = 7.77; **Figures 8I–K**), and abdominal distension had a reported case-fatality proportion of 25.93% (**Figure 8L**).

### 3.4 Signal detection at the high-level term and high-level group term levels

Adverse drug reactions were analyzed at the HLT and HLGT levels, with Sankey diagrams generated for the top ten Preferred Terms (PTs) within the gastrointestinal disorders SOCs.

In FAERS database, capecitabine demonstrated the strongest association with gastrointestinal neoplasms nec (**Figure 9A**). For gastrointestinal disorders SOCs, nausea and vomiting symptoms (HLT) and gastrointestinal signs and symptoms (HLGT) were most frequent (**Figure 9B**).

**Figure 9:**
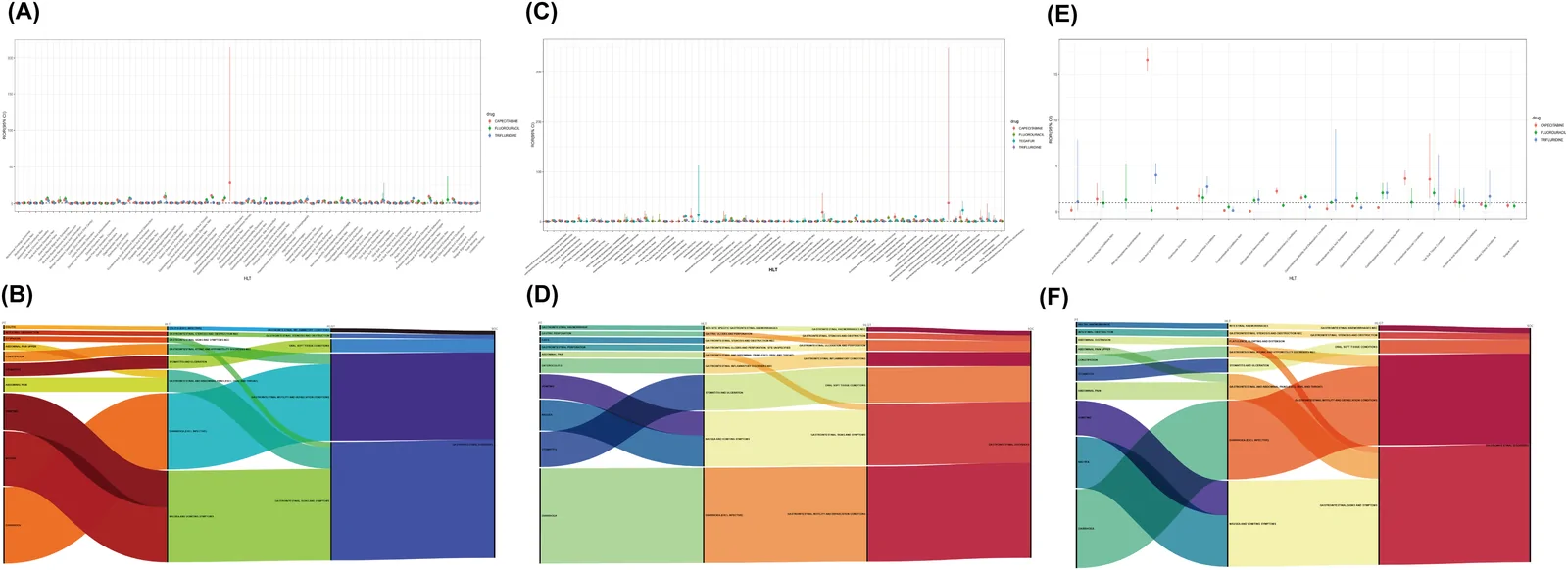
Disproportionality analyses at the HLT and HLGT levels. **(A)** HLT-level disproportionality analysis in FAERS. **(B)** PT–HLT–HLGT–SOC Sankey diagram for FAERS. **(C)** HLT-level disproportionality analysis in JADER. **(D)** PT–HLT–HLGT–SOC Sankey diagram for JADER. **(E)** HLT-level disproportionality analysis in CVARD. **(F)** PT–HLT–HLGT–SOC Sankey diagram for CVARD.

In JADER database, capecitabine demonstrated the strongest association with gastrointestinal neoplasms nec (**Figure 9C**). For gastrointestinal disorders SOCs, diarrhea(excl infective) (HLT) and gastrointestinal motility and defecation conditions (HLGT) were most frequent (**Figure 9D**).

In CVARD database, capecitabine demonstrated the strongest association with dental and gingival conditions (**Figure 9E**). For gastrointestinal disorders SOCs, nausea and vomiting symptoms (HLT) and gastrointestinal signs and symptoms (HLGT) were most frequent (**Figure 9F**).

### 3.5 Clinical-priority analysis

Reporting-signal prioritization scores were summarized separately by database for exploratory signal review. Death and hepatic failure were categorized as strong reporting-signal priority in FAERS, neutropenic sepsis in JADER, and death in CVARD (**Supplementary Tables S4**–**S6**). These categories do not constitute treatment recommendations or regulatory classifications.

### 3.6 TTO analysis

Among the included FAERS reports, evaluable time-to-onset (TTO) information was available for 5,108 of 19,012 5-FU reports (26.9%), 12,058 of 47,933 capecitabine reports (25.2%), and 2,661 of 9,013 trifluridine reports (29.5%). Because some reports contained more than one qualifying drug-start record, the corresponding numbers of evaluable TTO records were 5,413, 12,092, and 2,661, respectively. Only one evaluable tegafur record was identified; therefore, tegafur was included in the pooled descriptive analysis but was not fitted separately.

The median TTO was 22 days (interquartile range [IQR], 7–70 days) for 5-FU, 42 days (IQR, 16–110 days) for capecitabine, and 45 days (IQR, 20–97 days) for trifluridine. The pooled analysis included 20,167 evaluable TTO records and yielded a median TTO of 37 days (IQR, 13–98 days) (**Figures 10A, B**; **Supplementary Table S8**). These empirical distributions describe the timing of submitted reports and are not denominator-based time-to-event or cumulative-incidence estimates.

**Figure 10:**
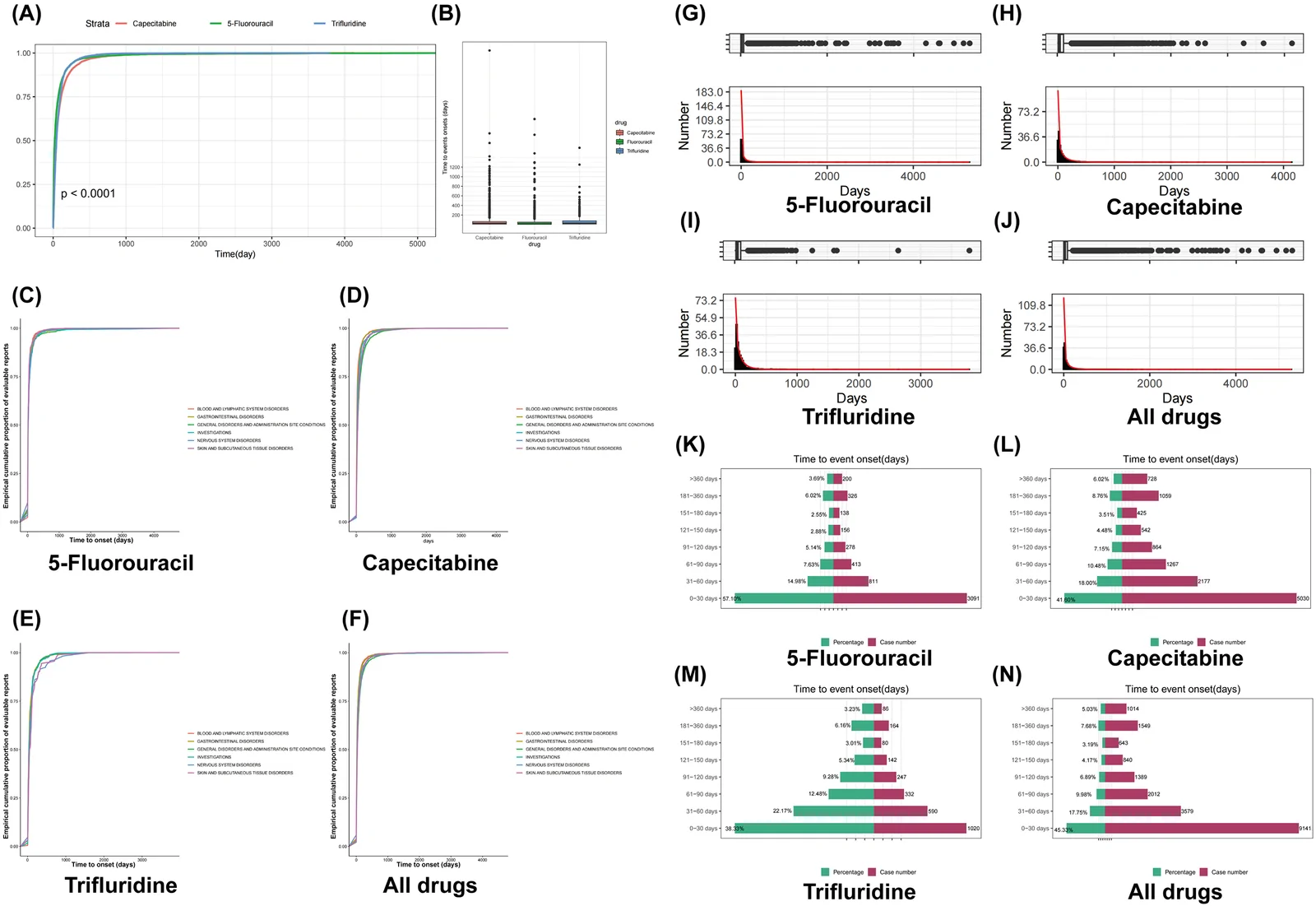
Time-to-onset patterns of fluoropyrimidine-associated adverse-event reports in FAERS. **(A)** Empirical cumulative distributions of TTO records according to fluoropyrimidine. **(B)** Distribution of TTO values across the three principal fluoropyrimidines. **(C–F)** Empirical cumulative distributions of TTO records according to gastrointestinal System Organ Class categories. **(G–J)** Observed TTO distributions and fitted Weibull curves for fluorouracil, capecitabine, trifluridine, and all fluoropyrimidines, respectively. **(K–N)** Numbers and proportions of TTO records within predefined reporting intervals. Drug-specific results were as follows: fluorouracil, n = 5,413, median TTO 22 days (IQR, 7–70 days), α = 50.25 (95% CI 47.89–52.61), and β = 0.602 (95% CI 0.591–0.613); capecitabine, n = 12,092, median TTO 42 days (IQR, 16–110 days), α = 81.94 (95% CI 79.75–84.13), and β = 0.706 (95% CI 0.697–0.714); trifluridine, n = 2,661, median TTO 45 days (IQR, 20–97 days), α = 75.18 (95% CI 71.63–78.72), and β = 0.853 (95% CI 0.830–0.876); and all fluoropyrimidines, n = 20,167, median TTO 37 days (IQR, 13–98 days), α = 71.80 (95% CI 70.26–73.33), and β = 0.684 (95% CI 0.677–0.690). Exact numbers of unique evaluable reports and TTO records, median values, IQRs, ranges, and Weibull estimates are provided in **Supplementary Table S8**. Priority-stratified findings are provided in **Supplementary Table S7**. These empirical distributions describe the timing of submitted FAERS reports and should not be interpreted as cumulative incidence. TTO analysis was limited to FAERS because complete onset-date information was unavailable in JADER and CVARD.

TTO distributions were also summarized for selected gastrointestinal System Organ Class categories (**Figures 10C–F**). Most evaluable TTO records were concentrated within the first month after treatment initiation (**Figures 10K–N**). Although a comparatively large number of records fell within the broad 181–360-day interval, this pattern should be interpreted in light of the unequal interval widths, possible longer or repeated exposure, retreatment, delayed reporting, incomplete reporting histories, and extreme observations. It was therefore not interpreted as evidence of a distinct late-toxicity peak.

Weibull modelling supported an early-reporting pattern for all three principal fluoropyrimidines (**Figures 10G-J**; **Supplementary Table S8**). The Weibull scale (α) and shape (β) estimates were 50.25 (95% confidence interval [CI], 47.89–52.61) and 0.602 (95% CI, 0.591–0.613), respectively, for 5-FU; 81.94 (95% CI, 79.75–84.13) and 0.706 (95% CI, 0.697–0.714) for capecitabine; and 75.18 (95% CI, 71.63–78.72) and 0.853 (95% CI, 0.830–0.876) for trifluridine. In the pooled analysis, α was 71.80 (95% CI, 70.26–73.33) and β was 0.684 (95% CI, 0.677–0.690). Because all shape parameters were below 1, reporting was more concentrated during the earlier period after treatment initiation and declined thereafter.

For gastrointestinal disorders, the median TTO was 22 days (IQR, 9–54 days) in the moderate-priority category and 22 days (IQR, 8–56 days) in the weak-priority category. The corresponding Weibull shape parameters were also below 1 (**Supplementary Table S7**). In a sensitivity analysis excluding five extreme records with TTO values exceeding 10,000 days, the pooled median TTO was 36 days (IQR, 13–98 days), and the Weibull shape parameter remained below 1 (β = 0.697, 95% CI, 0.691–0.704; **Supplementary Table S8**). This result was consistent with the early-reporting pattern observed in the primary analysis. Accordingly, the 181–360-day distribution was treated as exploratory and not as evidence of increasing event frequency among treated patients or a validated delayed-toxicity mechanism.

### 3.7 Sex-stratified analysis

Exploratory sex-stratified ROR analyses compared PT reporting between female and male reports (**Figure 11**). These analyses describe reporting disproportionality and do not estimate sex-specific event frequency or susceptibility.

**Figure 11:**
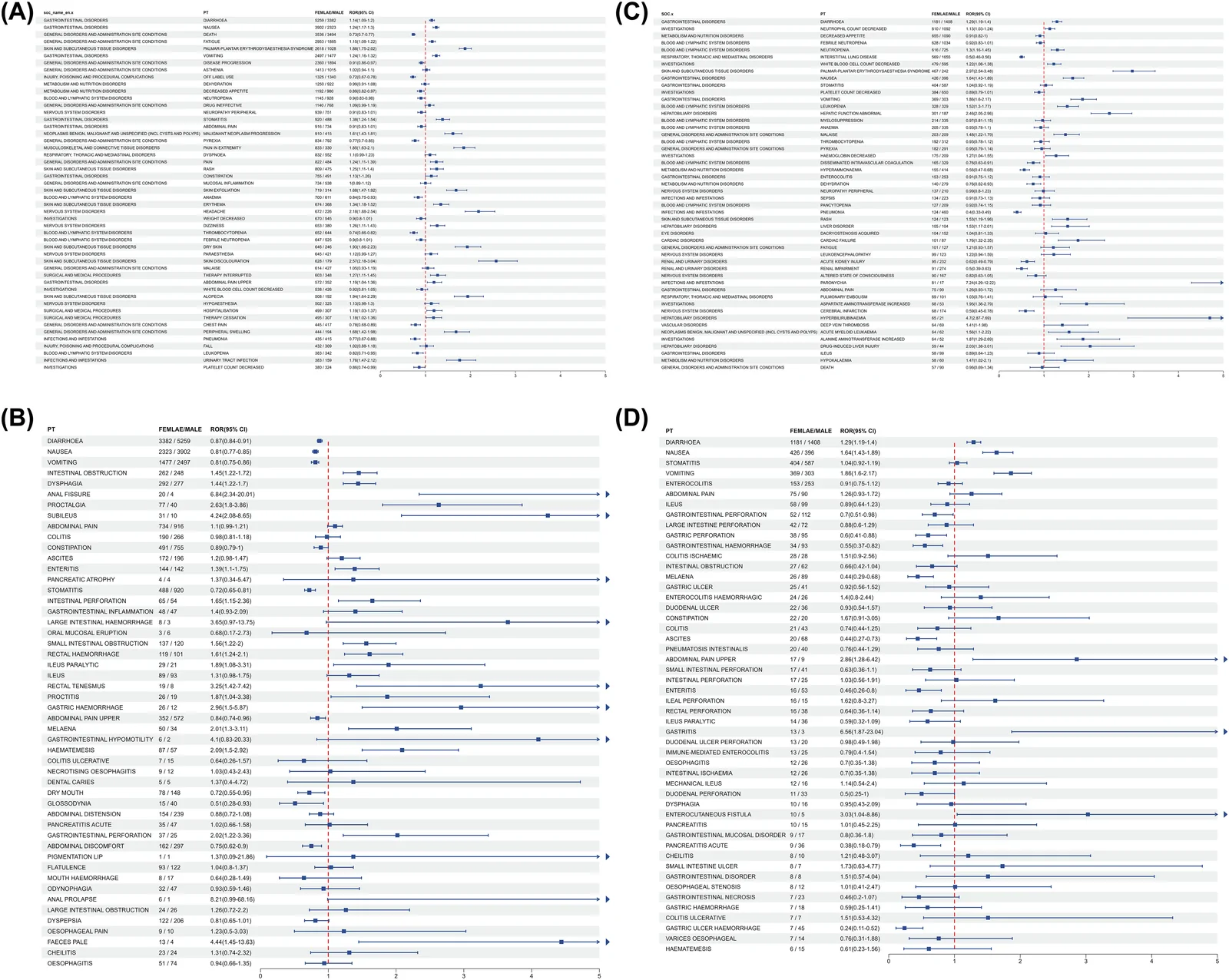
Exploratory sex-stratified reporting analyses at the SOC and PT levels. **(A, B)** FAERS sex-stratified analyses for all SOCs and gastrointestinal PTs. **(C, D)** corresponding JADER analyses. Results describe reporting disproportionality and not sex-specific event frequency or susceptibility.

In FAERS, several top PTs were more frequently reported in female reports. Skin discoloration showed a female-enriched reporting signal [ROR = 2.57 (95% CI 2.18-3.04)] (**Figure 11A**). Within gastrointestinal disorders, the estimate for anal prolapse was imprecise [ROR = 8.21 (95% CI 0.99-68.16)] and included the null value (**Figure 11B**).

In JADER, paronychia was enriched in female reports [ROR = 7.24 (95% CI 4.29-12.22)] and pneumonia in male reports [ROR = 0.40 (95% CI 0.33-0.49)] (**Figure 11C**). Within gastrointestinal disorders, gastritis was enriched in female reports [ROR = 6.56 (95% CI 1.87-23.04)] and gastric-ulcer hemorrhage in male reports [ROR = 0.24 (95% CI 0.11-0.52)] (**Figure 11D**). These are exploratory reporting differences.

Sex-stratified FAERS results were visualized using PRR in **Figure 12A** and ROR in **Figure 12B**. Palmar-plantar erythrodysaesthesia syndrome and coronary artery spasm met signal criteria in both strata; this does not imply equal event frequency or causal sex effects.

**Figure 12:**
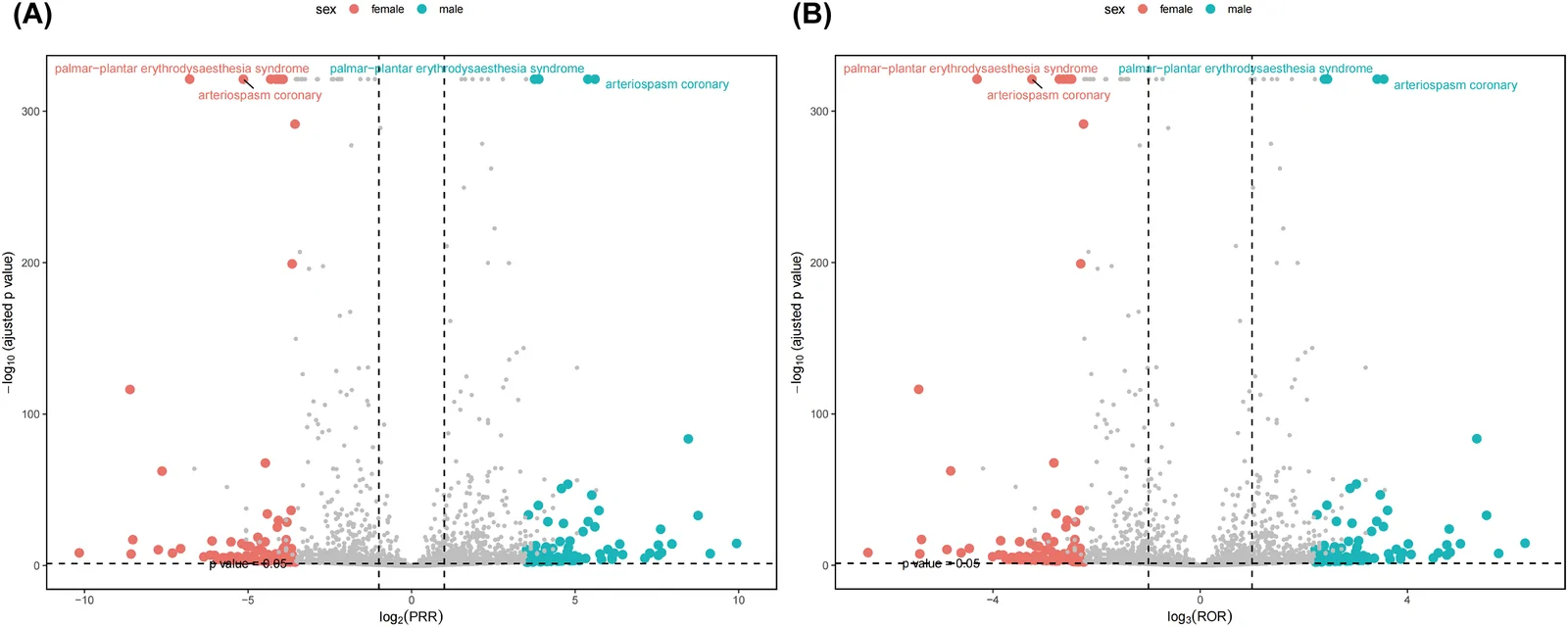
Sex-stratified PT disproportionality in FAERS. **(A)** PTs ranked by log2(PRR). **(B)** PTs ranked by log3(ROR).

### 3.8 *In vitro* experimental evidence of epithelial injury and epithelial inflammatory responses

Acute fluoropyrimidine exposure reduced epithelial cell viability and increased cell injury in both cell models. In GES-1 cells, CCK-8 viability decreased from 100.00 +/- 2.52% in the control group to 83.89 +/- 4.21%, 71.29 +/- 3.92%, and 72.05 +/- 3.33% after 5-FU, capecitabine, and trifluridine exposure, respectively (overall ANOVA P < 0.0001; Dunnett-adjusted P = 0.0014, P < 0.0001, and P < 0.0001 vs control). A similar pattern was observed in NCM460 cells, with viability decreasing to 68.43 +/- 5.94%, 56.02 +/- 3.96%, and 62.05 +/- 6.48% after the same treatments (overall ANOVA P < 0.0001; all Dunnett-adjusted P <= 0.0002 vs control). LDH release increased in parallel, particularly after capecitabine and trifluridine exposure, supporting direct epithelial injury (**Figure 2B**).

Oxidative stress and mitochondrial dysfunction were also observed. Acute ROS levels increased in GES-1 cells from 1.00 +/- 0.07 to 1.57 +/- 0.13, 2.39 +/- 0.38, and 1.74 +/- 0.10 after 5-FU, capecitabine, and trifluridine exposure, respectively (overall ANOVA P = 0.0003). In NCM460 cells, ROS increased from 1.00 +/- 0.05 to 1.61 +/- 0.14, 1.58 +/- 0.16, and 2.39 +/- 0.33, respectively (overall ANOVA P = 0.0002). Consistently, the JC-1 red/green ratio decreased in both GES-1 and NCM460 cells, indicating mitochondrial membrane depolarization (**Figure 2C**).

Under the 7-day repeated-exposure conditions, fluoropyrimidine treatment was associated with marked epithelial injury. In GES-1 cells, viability decreased to 78.55 +/- 10.23%, 51.81 +/- 13.10%, and 33.55 +/- 2.54% after 5-FU, capecitabine, and trifluridine treatment, respectively (overall ANOVA P < 0.0001). In NCM460 cells, viability decreased to 67.35 +/- 11.88%, 53.98 +/- 14.62%, and 38.49 +/- 7.13%, respectively (overall ANOVA P = 0.0009). Under the repeated-exposure conditions, LDH release and ROS accumulation increased, whereas JC-1 red/green ratios decreased across the fluoropyrimidine-treatment groups (**Figures 2D, E**).

qPCR showed lower barrier-related transcript abundance after fluoropyrimidine exposure: *TJP1* decreased to 0.67-, 0.45-, and 0.32-fold; *OCLN* to 0.83-, 0.39-, and 0.35-fold; and *CDH1* to 0.72-, 0.36-, and 0.23-fold after 5-FU, capecitabine, and trifluridine, respectively. Selected epithelial inflammatory-response genes (*IL6, TNF, IL1B, CXCL8, and CCL2*) and signaling-related genes (*TLR4, NFKB1, and NLRP3*) increased, as did extracellular-matrix-remodeling genes (*MMP9, COL1A1, and FN1*) (**Figure 2F**). These expression changes support an epithelial inflammatory response under the tested conditions but do not establish direct activation of TLR4, NF-κB, or NLRP3 signaling pathways.

### 3.9 Functional evidence of epithelial barrier impairment and cytokine release

FITC-dextran permeability differed across treatment groups in both GES-1 (F(3,8) = 7.895, P = 0.0089) and NCM460 (F(3,8) = 13.91, P = 0.0015) epithelial monolayers. Relative permeability increased from the vehicle-normalized value of 1.00 to 1.88, 2.20, and 2.34 after 5-FU, capecitabine, and trifluridine exposure, respectively, in GES-1 cells (Dunnett-adjusted P = 0.0483, 0.0106, and 0.0057) and to 1.65, 2.15, and 1.95, respectively, in NCM460 cells (adjusted P = 0.0230, 0.0008, and 0.0028) (**Figure 3A**). Because reduced cell viability and increased cellular injury were also observed under repeated-exposure conditions, the increased FITC-dextran passage may reflect both altered epithelial barrier integrity and cytotoxicity-associated loss of monolayer continuity.

Relative supernatant IL-6 levels also differed among treatment groups in GES-1 (F(3,8) = 10.46, P = 0.0038) and NCM460 (F(3,8) = 11.68, P = 0.0027) cultures. Compared with the vehicle-normalized value of 1.00, the mean relative IL-6 levels after 5-FU, capecitabine, and trifluridine exposure were 1.53, 1.93, and 1.78 in GES-1 cells (adjusted P = 0.0424, 0.0021, and 0.0059) and 1.71, 2.29, and 1.81 in NCM460 cells (adjusted P = 0.0299, 0.0010, and 0.0155) (**Figure 3B**).

Relative supernatant TNF-α levels differed among treatment groups in GES-1 (F(3,8) = 7.103, P = 0.0121) and NCM460 (F(3,8) = 22.23, P = 0.0003) cultures. The corresponding mean relative levels were 1.74, 2.02, and 1.92 in GES-1 cells (adjusted P = 0.0395, 0.0080, and 0.0142) and 1.52, 2.23, and 1.85 in NCM460 cells (adjusted P = 0.0253, 0.0001, and 0.0016) after 5-FU, capecitabine, and trifluridine exposure, respectively (**Figure 3C**). Together, these findings show that fluoropyrimidine exposure was associated with increased epithelial permeability and cytokine release under the tested conditions. Because the experiments used epithelial monocultures, they do not establish immune-cell activation or epithelial–immune interactions.

## 4 Discussion

### 4.1 Comparison with authoritative prescribing information

The FAERS, JADER, and CVARD reporting patterns were compared with DailyMed prescribing information for capecitabine, 5-FU, tegafur, and trifluridine/tipiracil (16, 18, 19). Gastrointestinal and hematological events were prominent in both the reporting analyses and existing labels (**Figures 6**–**8**; **Tables 2**–**7**). This qualitative concordance supports further signal evaluation but does not validate event frequency among treated populations or demographic susceptibility.

Tegafur-gastric perforation (ROR = 20.55) and trifluridine-intestinal obstruction (ROR = 7.77) showed strong reporting disproportionality (**Figures 8F, G**, **8J, K**). Reported case-fatality proportions for intestinal obstruction/colitis (14.46% in FAERS) and gastrointestinal hemorrhage (25.00% in JADER) indicate seriousness among submitted reports and do not estimate fatality among treated patients (**Figures 8D, H**).

Sex- and weight-related findings were treated as exploratory differences among submitted reports (**Table 1**; **Figures 11**, **12**). The FAERS TTO distribution was predominantly early, while the broad 181-360-day interval contained an exploratory late cluster (**Figures 10G–N**; **Supplementary Table S7**). These observations require denominator-based confirmation and should not be used to infer precision-dosing subgroups or a delayed population-level event pattern.

The present data do not support mandatory surveillance schedules, a >=12-month follow-up requirement, sex-specific preventive protocols, or a new 24-hour intervention classification. Clinicians should continue to follow established labeling and guidelines while the identified signals are evaluated in exposure-adjusted observational studies.

Likewise, the findings do not justify black-box-warning changes or new precision-dosing pathways. Confirmation should use exposure-adjusted pharmacoepidemiological studies, electronic health records, cancer registries, claims data, or prospective cohorts with adjudicated outcomes and treatment-duration information.

### 4.2 Epithelial-cell findings and pharmacological context

Dihydropyrimidine dehydrogenase, encoded by *DPYD*, is an established determinant of fluoropyrimidine metabolism and toxicity (20–23). These external pharmacogenetic data provide biological context, but they do not validate the database-specific demographic patterns or permit individual event-probability estimation from spontaneous reports.

The <50-kg category comprised a substantial proportion of submitted JADER reports with recorded weight, but missing weight data and the absence of an exposed comparator preclude an inference of increased toxicity or a new dosing recommendation. Future exposure-adjusted studies should evaluate body size, body-surface-area-normalized dose intensity, organ function, *DPYD* status, pharmacokinetics, and measured 5-FU exposure before determining whether dose modification or therapeutic drug monitoring is warranted.

Variants in *DPYD*, *TYMS*, and other pharmacokinetic or pharmacodynamic genes are plausible effect modifiers (24–27), but their contribution cannot be inferred from the present databases because genotype, dose, treatment duration, and exposure measurements were unavailable.

The observed sex-related reporting differences do not establish estrogen-mediated skin toxicity, androgen-related gastric vulnerability, or behavioral explanations. Reviews of hand-foot syndrome and studies of hormone-related keratinocyte biology provide background for future research (28–31), while literature on prostaglandin-dependent mucosal defense provides separate biological context (32–34). None of these sources establishes a mechanism for the present sex-stratified reporting differences, which remain exploratory.

The distribution of evaluable FAERS reports between days 181 and 360 should be interpreted cautiously. Unequal interval widths, prolonged or repeated treatment, retreatment, incomplete dates, extreme observations, and latency between event occurrence and report submission may contribute to the apparent late cluster. The 7-day repeated-exposure experiment does not reproduce or validate clinical reports occurring 6–12 months after treatment initiation. Mechanistic literature from other organ systems provides only indirect context for hypothesis generation (35).

The *in vitro* experiments provide epithelial-cell context for, but do not validate or explain, the gastrointestinal reporting signals. Acute 5-FU, capecitabine, and trifluridine exposure reduced viability, increased LDH release and ROS, and lowered the JC-1 red/green ratio in GES-1 and NCM460 cells, supporting epithelial injury, oxidative stress, and mitochondrial depolarization under the tested conditions (**Figures 2B, C**); related oxidative and mitochondrial observations have been described in intestinal and non-intestinal experimental systems (36, 37).

The 7-day repeated-exposure model produced greater injury than the acute protocol under the tested conditions (**Figures 2D, E**). It should be interpreted as a repeated-exposure model and not as experimental validation of the exploratory 181-360-day FAERS reporting distribution (**Figures 10G–N**; **Supplementary Table S7**).

The qPCR findings indicated altered expression of barrier-related, inflammatory-response, and signaling-related genes (**Figure 2F**). Increased supernatant IL-6 and TNF-α levels provided complementary evidence of an epithelial inflammatory response (**Figures 3B, C**). However, these transcriptional and cytokine changes do not establish direct activation of the TLR4, NF-κB, or NLRP3 signaling pathways. The findings remain confined to epithelial-cell models and should not be interpreted as evidence of immune-cell activation.

The present study extends the transcriptional observations by showing that altered barrier-related gene expression (**Figure 2F**) was accompanied by increased FITC-dextran permeability (**Figure 3A**), supporting functional epithelial barrier impairment. Because western blotting and junction-localization assays were not performed, the study does not establish reduced zonula occludens-1 (ZO-1), Occludin, or epithelial cadherin (E-cadherin) protein abundance or altered junctional localization.

Although relative supernatant IL-6 and TNF-α levels increased (**Figures 3B, C**), epithelial monoculture models cannot directly evaluate immune-cell activation, recruitment, or epithelial–immune interactions. Complementary approaches, including immunofluorescence, transepithelial electrical resistance measurements, western blotting, polarized co-culture systems, and organoid models, are needed to further characterize junctional alterations and epithelial–immune crosstalk.

### 4.3 Limitations

This study is limited by the passive and heterogeneous nature of FAERS, JADER, and CVARD, including underreporting, stimulated and delayed reporting, duplicate or follow-up reports, indication and notoriety biases, differential market use, missing exposure denominators, and incomplete clinical covariates (38–40). Accordingly, all disproportionality, subgroup, and time-to-onset findings represent reporting associations among submitted reports; they do not estimate incidence, relative risk, or causal effects in treated populations. Cross-database demographic differences are descriptive only. TTO was evaluable only in FAERS and may be distorted by incomplete dates, unequal time bins, treatment duration, repeated cycles, extreme intervals, and event-to-report latency. The epithelial monocultures do not model immune-cell activation, recruitment, metabolism by liver or tumor tissue, or the full clinical formulation. Capecitabine requires multistep bioactivation whose capacity was not measured in GES-1 or NCM460 cells; trifluridine was tested without tipiracil; and tegafur was not included *in vitro*. The permeability and ELISA experiments provide functional and secreted-cytokine evidence, but western blotting, junctional immunofluorescence, transepithelial electrical resistance, polarized co-culture, and organoid validation were not performed. These limitations require exposure-adjusted clinical studies and complementary experiments using active metabolites or metabolically competent models.

## 5 Conclusion

This study identified fluoropyrimidine-associated gastrointestinal reporting signals across FAERS, JADER, and CVARD (**Figures 6**–**9**; **Tables 2**–**7**). FAERS TTO analysis showed an overall early-reporting pattern; the report distribution between days 181 and 360 was exploratory and does not establish delayed event occurrence among treated patients (**Figure 10**; **Supplementary Table S7**).

Across the GES-1 and NCM460 epithelial-cell experiments, fluoropyrimidine exposure was associated with lower viability, greater LDH release and ROS accumulation, mitochondrial depolarization, and reduced expression of barrier-related genes (**Figures 2B–F**). In the representative-treatment experiments, FITC-dextran permeability and relative supernatant IL-6 and TNF-α levels increased (**Figures 3A–C**). Together, these data support epithelial injury, functional barrier impairment, and epithelial inflammatory responses under the tested conditions, but they do not establish junctional protein loss or immune-cell activation. The direct *in vitro* exposure used trifluridine without tipiracil and should not be equated with the clinical combination. The pharmacovigilance signals are hypothesis-generating and require confirmation in exposure-adjusted observational studies or prospective cohorts.

## Data Availability

The original contributions presented in the study are included in the article/**Supplementary Material**. Further inquiries can be directed to the corresponding author.

## Glossary

5-FU: 5-fluorouracil
ADE: Adverse Drug Event
AE: adverse event
AER: Adverse Event Report
BCPNN: Bayesian Confidence Propagation Neural Network
CCK-8: Cell Counting Kit-8
CI: Confidence Interval
CVARD: Canada Vigilance Adverse Reaction Database
DEMO: Demographic and administrative information
DME: Designated Medical Event
DMSO: dimethyl sulfoxide
DPD: Dihydropyrimidine Dehydrogenase
DRUG: drug information
E-cadherin: epithelial cadherin
EBGM: Empirical Bayes Geometric Mean
ELISA: enzyme-linked immunosorbent assay
FAERS: US Food and Drug Administration Adverse Event Reporting System
FDA: Food and Drug Administration
FITC: fluorescein isothiocyanate
HIST: medical history
HLGT: High-Level Group Term
HLT: High-Level Term
IL-6: interleukin-6
IME: Important Medical Event
INDI: Indications for use
IQR: interquartile range
JADER: Japanese Adverse Drug Event Report database
LDH: lactate dehydrogenase
MedDRA: Medical Dictionary for Regulatory Activities
OUTC: Patient outcomes
PRR: Proportional Reporting Ratio
PT: Preferred Term
qPCR: quantitative real-time polymerase chain reaction
REAC: Adverse reaction data
ROR: Reporting Odds Ratio
ROS: reactive oxygen species
RPSR: Reporting sources
SD: standard deviation
SOC: System Organ Class
SRS: Spontaneous Reporting System
THER: Drug therapy start and end dates
TNF-α: tumor necrosis factor alpha
TTO: time to onset
UIMA: Unstructured Information Management Architecture
ZO-1: zonula occludens-1
