# Supplementary material for: Fluoropyrimidine-associated gastrointestinal toxicity: pharmacovigilance reporting signals and in vitro evidence of epithelial injury, barrier impairment, and inflammatory responses
Source: Front Immunol. 2026 Sep 29;17:1934652. doi: 10.3389/fimmu.2026.1934652 (PMC13658617; doi:10.3389/fimmu.2026.1934652)
Supplement: Supplementary file 1 [file DataSheet1.docx]

Table S1. Two-by-two contingency table used for disproportionality analysis.

|  | Target adverse event | All other adverse events | Total |
| --- | --- | --- | --- |
| Reports involving the target fluoropyrimidine | a | b | a+b |
| Reports involving all other drugs in the same database | c | d | c+d |
| Total | a+c | b+d | N=a+b+c+d |

Table S2. Equations and prespecified criteria for the four disproportionality methods.

| Method | Equation | Prespecified signal criterion |
| --- | --- | --- |
| ROR | ROR= $\frac{a/c}{b/d}$ | a$\geq$3 and 95% CI (lower limit) > 1 |
|  | *SE*(*ln*ROR)=$\sqrt{\frac{1}{a}+\frac{1}{b}+\frac{1}{c}+\frac{1}{d}}$ |  |
|  | 95%*CI*=$e^{ln(ROR)\pm1.96\sqrt{\frac{1}{a}+\frac{1}{b}+\frac{1}{c}+\frac{1}{d}}}$ |  |
| PRR | PRR=$\frac{a/(a+b)}{c/(c+d)}$ | a$\geq$3 and 95% CI (lower limit) > 1 |
|  | *SE*(*ln*PRR)=$\sqrt{\frac{1}{a}-\frac{1}{a+b}+\frac{1}{c}-\frac{1}{c+d}}$ |  |
|  | 95%*CI*=$e^{ln(PRR)\pm1.96\sqrt{\frac{1}{a}-\frac{1}{a+b}+\frac{1}{c}-\frac{1}{c+d}}}$ |  |
| BCPNN | IC=${log}_{2}\frac{p(x,y)}{p\left( x \right)p(y)}$=${log}_{2}\frac{a(a+b+c+d)}{(a+b)(a+c)}$ | IC025 > 0 |
|  | IC-2SD=E(IC)-2$\sqrt{V(IC)}$ |  |
| EBGM | EBGM=$\frac{a/(a+b+c+d)}{(a+c)/(a+b)}$ | EBGM05 > 2 |
|  | 95%CI=$e^{ln(EBGM)\pm1.96\sqrt{\frac{1}{a}+\frac{1}{b}+\frac{1}{c}+\frac{1}{d}}}$ |  |

Table S3. Criteria and scores used to prioritize adverse-event reporting signals.

| Criterion | 2 points | 1 point | 0 point |
| --- | --- | --- | --- |
| Reporting proportion (target-event reports among evaluated reports) | > 10% | 1–10% | 0–1% |
| Signal consistency across the four disproportionality methods | 4 of 4 | 3 of 4 | ≤2 of 4 |
| Reported case-fatality proportion (reports with death outcome among reports for the PT) | > 50% | 25–50% | < 25% |
| Serious-event classification | Designated medical event (DME) | Important medical event (IME) | None |

Table S4. Prioritization scores for fluoropyrimidine-associated preferred-term reporting signals in FAERS (top 50).

| **PT** | **Reporting proportion score** | **Signal consistency score** | **Reported case-fatality proportion score** | **Serious-event classification score** | **Overall score** | **Reporting-signal priority** |
| --- | --- | --- | --- | --- | --- | --- |
| death | 1 | 2 | 2 | 1 | 6 | strong priority |
| hepatic failure | 0 | 2 | 2 | 2 | 6 | strong priority |
| pancytopenia | 0 | 2 | 1 | 2 | 5 | moderate priority |
| septic shock | 0 | 2 | 2 | 1 | 5 | moderate priority |
| neutropenic sepsis | 0 | 2 | 1 | 2 | 5 | moderate priority |
| intestinal perforation | 0 | 2 | 1 | 2 | 5 | moderate priority |
| disseminated intravascular coagulation | 0 | 2 | 2 | 1 | 5 | moderate priority |
| colon cancer metastatic | 0 | 2 | 2 | 1 | 5 | moderate priority |
| sudden death | 0 | 2 | 2 | 1 | 5 | moderate priority |
| neutropenic colitis | 0 | 2 | 1 | 2 | 5 | moderate priority |
| cachexia | 0 | 2 | 2 | 1 | 5 | moderate priority |
| metastases to meninges | 0 | 2 | 2 | 1 | 5 | moderate priority |
| rectal cancer metastatic | 0 | 2 | 2 | 1 | 5 | moderate priority |
| lymphangiosis carcinomatosa | 0 | 2 | 2 | 1 | 5 | moderate priority |
| malignant pleural effusion | 0 | 2 | 2 | 1 | 5 | moderate priority |
| neutropenic infection | 0 | 2 | 1 | 2 | 5 | moderate priority |
| necrotising colitis | 0 | 2 | 2 | 1 | 5 | moderate priority |
| megacolon | 0 | 2 | 2 | 1 | 5 | moderate priority |
| ear malformation | 0 | 2 | 2 | 1 | 5 | moderate priority |
| oesophageal cancer metastatic | 0 | 2 | 2 | 1 | 5 | moderate priority |
| hyperleukocytosis | 0 | 2 | 2 | 1 | 5 | moderate priority |
| metastatic gastric cancer | 0 | 2 | 2 | 1 | 5 | moderate priority |
| hepatorenal failure | 0 | 2 | 2 | 1 | 5 | moderate priority |
| congenital pulmonary airway malformation | 0 | 2 | 2 | 1 | 5 | moderate priority |
| intestinal infarction | 0 | 2 | 2 | 1 | 5 | moderate priority |
| abdominal compartment syndrome | 0 | 2 | 2 | 1 | 5 | moderate priority |
| colon cancer stage iv | 0 | 2 | 2 | 1 | 5 | moderate priority |
| central nervous system haemorrhage | 0 | 2 | 2 | 1 | 5 | moderate priority |
| gastrointestinal ischaemia | 0 | 2 | 2 | 1 | 5 | moderate priority |
| gastrointestinal mucosal necrosis | 0 | 2 | 2 | 1 | 5 | moderate priority |
| tumour fistulisation | 0 | 2 | 2 | 1 | 5 | moderate priority |
| hepatic infarction | 0 | 2 | 1 | 2 | 5 | moderate priority |
| oesophageal squamous cell carcinoma | 0 | 2 | 2 | 1 | 5 | moderate priority |
| pulmonary tumour thrombotic microangiopathy | 0 | 2 | 2 | 1 | 5 | moderate priority |
| glossopharyngeal nerve paralysis | 0 | 2 | 2 | 1 | 5 | moderate priority |
| thrombosis mesenteric vessel | 0 | 2 | 2 | 1 | 5 | moderate priority |
| tumour ulceration | 0 | 2 | 2 | 1 | 5 | moderate priority |
| perineal ulceration | 0 | 2 | 2 | 1 | 5 | moderate priority |
| rectosigmoid cancer stage iii | 0 | 2 | 2 | 1 | 5 | moderate priority |
| rectal cancer stage iv | 0 | 2 | 2 | 1 | 5 | moderate priority |
| visceral venous thrombosis | 0 | 2 | 2 | 1 | 5 | moderate priority |
| oesophageal ulcer perforation | 0 | 2 | 2 | 1 | 5 | moderate priority |
| metastases to gastrointestinal tract | 0 | 2 | 2 | 1 | 5 | moderate priority |
| cerebral haemorrhage neonatal | 0 | 2 | 2 | 1 | 5 | moderate priority |
| gastrooesophageal cancer | 0 | 2 | 2 | 1 | 5 | moderate priority |
| mesenteric vascular occlusion | 0 | 2 | 2 | 1 | 5 | moderate priority |
| conjunctival erosion | 0 | 2 | 2 | 1 | 5 | moderate priority |
| middle cerebral artery stroke | 0 | 2 | 2 | 1 | 5 | moderate priority |
| metastases to salivary gland | 0 | 2 | 2 | 1 | 5 | moderate priority |
| disease progression | 1 | 2 | 1 | 0 | 4 | moderate priority |

Table S5. Prioritization scores for fluoropyrimidine-associated preferred-term reporting signals in JADER (top 50).

| **PT** | **Reporting proportion score** | **Signal consistency score** | **Reported case-fatality proportion score** | **Serious-event classification score** | **Overall score** | **Reporting-signal priority** |
| --- | --- | --- | --- | --- | --- | --- |
| neutropenic sepsis | 0 | 2 | 2 | 2 | 6 | strong priority |
| febrile neutropenia | 1 | 2 | 0 | 2 | 5 | moderate priority |
| disseminated intravascular coagulation | 1 | 2 | 1 | 1 | 5 | moderate priority |
| necrotising colitis | 0 | 2 | 2 | 1 | 5 | moderate priority |
| thrombosis mesenteric vessel | 0 | 2 | 2 | 1 | 5 | moderate priority |
| pelvic infection | 0 | 2 | 2 | 1 | 5 | moderate priority |
| hepatic amoebiasis | 0 | 2 | 2 | 1 | 5 | moderate priority |
| aortic injury | 0 | 2 | 2 | 1 | 5 | moderate priority |
| neutropenia | 1 | 2 | 0 | 1 | 4 | moderate priority |
| leukopenia | 1 | 2 | 0 | 1 | 4 | moderate priority |
| myelosuppression | 1 | 2 | 0 | 1 | 4 | moderate priority |
| thrombocytopenia | 1 | 2 | 0 | 1 | 4 | moderate priority |
| myelodysplastic syndrome | 0 | 2 | 1 | 1 | 4 | moderate priority |
| acute myeloid leukaemia | 0 | 2 | 1 | 1 | 4 | moderate priority |
| hepatic failure | 0 | 0 | 2 | 2 | 4 | moderate priority |
| neutropenic infection | 0 | 2 | 0 | 2 | 4 | moderate priority |
| thrombotic thrombocytopenic purpura | 0 | 0 | 2 | 2 | 4 | moderate priority |
| pulmonary fibrosis | 0 | 1 | 1 | 2 | 4 | moderate priority |
| lymphangiosis carcinomatosa | 0 | 1 | 2 | 1 | 4 | moderate priority |
| mesenteric artery thrombosis | 0 | 2 | 1 | 1 | 4 | moderate priority |
| colorectal cancer metastatic | 0 | 2 | 1 | 1 | 4 | moderate priority |
| aorto-oesophageal fistula | 0 | 2 | 1 | 1 | 4 | moderate priority |
| neutropenic colitis | 0 | 1 | 1 | 2 | 4 | moderate priority |
| tracheo-oesophageal fistula | 0 | 2 | 1 | 1 | 4 | moderate priority |
| bladder perforation | 0 | 2 | 1 | 1 | 4 | moderate priority |
| oesophageal fistula | 0 | 2 | 1 | 1 | 4 | moderate priority |
| chronic myelomonocytic leukaemia | 0 | 2 | 1 | 1 | 4 | moderate priority |
| myelodysplastic syndrome transformation | 0 | 2 | 1 | 1 | 4 | moderate priority |
| gastrointestinal mucosal necrosis | 0 | 2 | 1 | 1 | 4 | moderate priority |
| haemorrhagic tumour necrosis | 0 | 2 | 1 | 1 | 4 | moderate priority |
| amoebic colitis | 0 | 2 | 1 | 1 | 4 | moderate priority |
| amoebic dysentery | 0 | 1 | 2 | 1 | 4 | moderate priority |
| neuroendocrine carcinoma | 0 | 2 | 1 | 1 | 4 | moderate priority |
| diarrhoea | 1 | 2 | 0 | 0 | 3 | moderate priority |
| neutrophil count decreased | 1 | 2 | 0 | 0 | 3 | moderate priority |
| decreased appetite | 1 | 2 | 0 | 0 | 3 | moderate priority |
| white blood cell count decreased | 1 | 2 | 0 | 0 | 3 | moderate priority |
| stomatitis | 1 | 2 | 0 | 0 | 3 | moderate priority |
| nausea | 1 | 2 | 0 | 0 | 3 | moderate priority |
| palmar-plantar erythrodysaesthesia syndrome | 1 | 2 | 0 | 0 | 3 | moderate priority |
| vomiting | 1 | 2 | 0 | 0 | 3 | moderate priority |
| hyperammonaemia | 1 | 2 | 0 | 0 | 3 | moderate priority |
| enterocolitis | 0 | 2 | 0 | 1 | 3 | moderate priority |
| leukoencephalopathy | 0 | 2 | 0 | 1 | 3 | moderate priority |
| septic shock | 0 | 1 | 1 | 1 | 3 | moderate priority |
| gastrointestinal perforation | 0 | 2 | 0 | 1 | 3 | moderate priority |
| gastric perforation | 0 | 2 | 0 | 1 | 3 | moderate priority |
| second primary malignancy | 0 | 2 | 0 | 1 | 3 | moderate priority |
| hyperbilirubinaemia | 0 | 2 | 0 | 1 | 3 | moderate priority |
| acute respiratory distress syndrome | 0 | 0 | 2 | 1 | 3 | moderate priority |

Table S6. Prioritization scores for fluoropyrimidine-associated preferred-term reporting signals in CVARD (top 50).

| **PT** | **Reporting proportion score** | **Signal consistency score** | **Reported case-fatality proportion score** | **Serious-event classification score** | **Overall score** | **Reporting-signal priority** |
| --- | --- | --- | --- | --- | --- | --- |
| death | 1 | 2 | 2 | 1 | 6 | strong priority |
| febrile neutropenia | 1 | 2 | 0 | 2 | 5 | moderate priority |
| ventricular fibrillation | 0 | 2 | 1 | 2 | 5 | moderate priority |
| myeloid leukaemia | 0 | 2 | 2 | 1 | 5 | moderate priority |
| bone marrow failure | 0 | 2 | 1 | 2 | 5 | moderate priority |
| rectal cancer metastatic | 0 | 2 | 2 | 1 | 5 | moderate priority |
| cardiac death | 0 | 2 | 2 | 1 | 5 | moderate priority |
| colon cancer metastatic | 0 | 2 | 2 | 1 | 5 | moderate priority |
| adenocarcinoma pancreas | 0 | 2 | 2 | 1 | 5 | moderate priority |
| malignant neoplasm progression | 1 | 2 | 0 | 1 | 4 | moderate priority |
| neutropenia | 1 | 2 | 0 | 1 | 4 | moderate priority |
| neuropathy peripheral | 1 | 2 | 0 | 1 | 4 | moderate priority |
| intestinal perforation | 0 | 2 | 0 | 2 | 4 | moderate priority |
| hepatic failure | 0 | 1 | 1 | 2 | 4 | moderate priority |
| ileus | 0 | 2 | 1 | 1 | 4 | moderate priority |
| thrombotic thrombocytopenic purpura | 0 | 2 | 0 | 2 | 4 | moderate priority |
| gastrointestinal perforation | 0 | 2 | 1 | 1 | 4 | moderate priority |
| ureteric obstruction | 0 | 2 | 1 | 1 | 4 | moderate priority |
| hypertensive heart disease | 0 | 2 | 2 | 0 | 4 | moderate priority |
| malignant pleural effusion | 0 | 2 | 1 | 1 | 4 | moderate priority |
| colorectal cancer recurrent | 0 | 2 | 1 | 1 | 4 | moderate priority |
| haemorrhagic infarction | 0 | 2 | 1 | 1 | 4 | moderate priority |
| vascular stent thrombosis | 0 | 2 | 1 | 1 | 4 | moderate priority |
| assisted suicide | 0 | 2 | 2 | 0 | 4 | moderate priority |
| disease progression | 1 | 2 | 0 | 0 | 3 | moderate priority |
| diarrhoea | 1 | 2 | 0 | 0 | 3 | moderate priority |
| neoplasm progression | 1 | 2 | 0 | 0 | 3 | moderate priority |
| palmar-plantar erythrodysaesthesia syndrome | 1 | 2 | 0 | 0 | 3 | moderate priority |
| breast cancer | 0 | 2 | 0 | 1 | 3 | moderate priority |
| colorectal cancer | 0 | 2 | 0 | 1 | 3 | moderate priority |
| breast cancer metastatic | 0 | 2 | 0 | 1 | 3 | moderate priority |
| metastases to liver | 0 | 2 | 0 | 1 | 3 | moderate priority |
| pulmonary embolism | 0 | 2 | 0 | 1 | 3 | moderate priority |
| cardiomyopathy | 0 | 2 | 0 | 1 | 3 | moderate priority |
| thrombosis | 0 | 2 | 0 | 1 | 3 | moderate priority |
| metastases to bone | 0 | 2 | 0 | 1 | 3 | moderate priority |
| myocarditis | 0 | 2 | 0 | 1 | 3 | moderate priority |
| metastases to central nervous system | 0 | 2 | 0 | 1 | 3 | moderate priority |
| neurotoxicity | 0 | 2 | 0 | 1 | 3 | moderate priority |
| cardiotoxicity | 0 | 2 | 0 | 1 | 3 | moderate priority |
| ascites | 0 | 2 | 0 | 1 | 3 | moderate priority |
| metastases to lung | 0 | 2 | 0 | 1 | 3 | moderate priority |
| cytopenia | 0 | 2 | 0 | 1 | 3 | moderate priority |
| metastases to lymph nodes | 0 | 2 | 0 | 1 | 3 | moderate priority |
| arteriospasm coronary | 0 | 2 | 0 | 1 | 3 | moderate priority |
| pancytopenia | 0 | 1 | 0 | 2 | 3 | moderate priority |
| acute myocardial infarction | 0 | 2 | 0 | 1 | 3 | moderate priority |
| myelosuppression | 0 | 2 | 0 | 1 | 3 | moderate priority |
| metastasis | 0 | 2 | 0 | 1 | 3 | moderate priority |
| cardiogenic shock | 0 | 2 | 0 | 1 | 3 | moderate priority |

Table S7. Time-to-onset analysis of reports classified as moderate or weak reporting-signal priority in FAERS.

| Prioritization | TTO (days) |  |  | Weibull distribution |  |  |  | Pattern classification |
| --- | --- | --- | --- | --- | --- | --- | --- | --- |
|  | Cases |  |  | Scale parameter |  | Shape parameter |  |  |
|  | n | Median (IQR) | Min–max | α | 95% CI | β | 95% CI |  |
| Moderate | 6891 | 22 (9–54) | 1–20,829 | 44.62 | (42.97–46.26) | 0.68 | (0.67–0.70) | Early-reporting pattern |
| Weak | 2698 | 22 (8–56) | 1–20,829 | 45.43 | (42.69–48.16) | 0.66 | (0.65–0.68) | Early-reporting pattern |

Table S8. Drug-specific time-to-onset distributions and Weibull estimates for fluoropyrimidine-associated reports in FAERS

| Drug | Total included reports | Unique reports with evaluable TTO, n (%) | Evaluable TTO records | Median (IQR), days | Range, days | Weibull α (95% CI) | Weibull β (95% CI) | Reporting pattern |
| --- | --- | --- | --- | --- | --- | --- | --- | --- |
| Fluorouracil | 19,012 | 5,108 (26.9%) | 5,413 | 22 (7–70) | 1–10,221 | 50.25 (47.89–52.61) | 0.602 (0.591–0.613) | Early |
| Capecitabine | 47,933 | 12,058 (25.2%) | 12,092 | 42 (16–110) | 1–20,829 | 81.94 (79.75–84.13) | 0.706 (0.697–0.714) | Early |
| Trifluridine | 9,013 | 2,661 (29.5%) | 2,661 | 45 (20–97) | 1–3,788 | 75.18 (71.63–78.72) | 0.853 (0.830–0.876) | Early |
| All fluoropyrimidines | 75,962 | 19,828 (26.1%) | 20,167 | 37 (13–98) | 1–20,829 | 71.80 (70.26–73.33) | 0.684 (0.677–0.690) | Early |
